# Barriers and facilitators to the use of virtual wards: a systematic review of the qualitative evidence

**DOI:** 10.1093/intqhc/mzaf065

**Published:** 2025-07-18

**Authors:** Sara Cucurachi, Sinéad Lydon, Laura Louise Moens, Tanja Manser, Paul O’Connor

**Affiliations:** Department of General Practice, School of Medicine, University of Galway, 1 Distillery Road, Galway, Co. Galway, H91 TK33, Ireland; Department of General Practice, School of Medicine, University of Galway, 1 Distillery Road, Galway, Co. Galway, H91 TK33, Ireland; Irish Centre for Applied Patient Safety and Simulation, School of Medicine, University of Galway, Newcastle Road, Galway, H91 YR71, Ireland; School of Applied Psychology, University of Applied Sciences and Arts Northwestern Switzerland (FHNW), Riggenbachstrasse 16, Olten, 4600, Switzerland; Graduate School for Health Sciences, University of Bern, Bühlplatz 5, Bern, 3012, Switzerland; School of Applied Psychology, University of Applied Sciences and Arts Northwestern Switzerland (FHNW), Riggenbachstrasse 16, Olten, 4600, Switzerland; Department of General Practice, School of Medicine, University of Galway, 1 Distillery Road, Galway, Co. Galway, H91 TK33, Ireland; Irish Centre for Applied Patient Safety and Simulation, School of Medicine, University of Galway, Newcastle Road, Galway, H91 YR71, Ireland

**Keywords:** Virtual Wards, Barriers, Facilitators, Home-based Care, Qualitative Methods, Qualitative Evidence, Behavioral change models

## Abstract

**Background:**

Virtual wards offer an alternative to traditional inpatient care, delivering acute care, monitoring, and treatment at home to prevent hospital admissions or facilitate early discharge. The aim of our qualitative systematic review was to understand the barriers to and facilitators for the successful implementation and sustainability of virtual wards from the perspective of any involved stakeholder, using behavioural change models.

**Methods:**

The review protocol was registered on PROSPERO (CRD42024519627). The following databases were searched: Medline, EMBASE, CINAHL, PsycINFO, and Academic Search Complete. A three-stage deductive content analysis, as recommended for applying the COM-B (Capability, Opportunity, and Motivation–Behaviour) model and TDF (Theoretical Domains Framework) to qualitative data, was conducted to categorize and map the barriers and facilitators to virtual wards identified in the included studies, using the TDF domains as a guiding framework.

**Results:**

Searches initially identified 7489 articles. Sixteen studies met the inclusion criteria. Common barriers for patients and family members were a lack of language skills, technical skills, and medical knowledge. Caregivers were also required to take on significant medical responsibilities, while patients had to remain self-motivated. The introduction of appropriate training was seen as a valuable facilitator. Healthcare providers faced numerous technological barriers that had the potential to affect care delivery. Strong leadership was an essential facilitator for effective care coordination in virtual wards. From a healthcare system perspective, the availability of resources — such as staffing, equipment, and funding —along with standardized protocols, is crucial for the successful implementation of virtual wards.

**Conclusions:**

Virtual wards can ease hospital capacity issues and support the delivery of safe and effective care in patients’ own homes. However, to realize this potential, we must understand the barriers to, and facilitators of, the use and successful implementation of virtual wards for patients, carers, and healthcare professionals. This understanding will allow targeted strategies and interventions to be developed to support both the delivery and receipt of care on virtual wards.

## Introduction

Virtual wards seek to mirror the mechanisms, staffing, and daily operations of a traditional hospital ward, without the need for a physical facility. In essence, virtual wards function as a multidisciplinary case management model that usually provides short-term transitional care [1]. The aim of this emerging model of care is to reduce non-elective secondary care usage through early discharge, by allowing patients to continue their recovery at home, and by preventing admissions or readmission to the hospital [2].

Systematic reviews have identified a positive impact of virtual wards across a range of metrics including patient safety, quality of care, and cost-effectiveness for the healthcare system [3]. For example, virtual wards have been associated with reduced patient mortality [4] and lower readmission rates [5]. In addition, care provided through virtual wards has been linked to improvements in health-related quality of life, such as reductions in negative emotions and physical discomfort [6]. One such review, by Chauhan *et al*. [3], specifically examined mortality and hospital readmissions among patients receiving virtual ward transitional care compared to those receiving usual post-discharge care. The authors found that virtual wards were associated with fewer emergency department visits, shorter hospital readmission stays, and reduced mortality. These outcomes also translated into significant cost savings for the healthcare system. Although these systematic reviews offer valuable quantitative data on the impact of virtual wards, they provide limited insight into why virtual wards are effective and fail to capture the lived experiences of those delivering or receiving care in these settings. Yet, capturing these experiences is essential for designing services that are not only clinically effective but also practical, acceptable, and responsive to the needs of both patients and healthcare professionals. Such insights are best gathered through qualitative research methods [7].

Qualitative data can ‘add value by providing decision-makers with additional evidence to improve understanding of intervention complexity, contextual variations, implementation, and stakeholder preferences and experiences’ [8]. Synthesizing qualitative data on the impact of virtual wards can provide critical insights into the barriers and enablers of their successful implementation and long-term sustainability. For instance, studies that have employed qualitative methodologies—such as interviews, focus groups, and questionnaires—have highlighted the importance of technical knowledge [9], trust between patients and providers [10], trust among members of multidisciplinary teams [11], and access to adequate resources [12] as key factors shaping the effectiveness of virtual ward models. A qualitative systematic review will allow researchers to synthesize these findings from multiple studies offering a comprehensive overview of the challenges and enablers related to the successful implementation and sustainability of virtual wards and informing practical, evidence-based recommendations.

The aim of our qualitative systematic review is to synthesize studies that have explored the barriers and facilitators to virtual wards from the perspectives of patients, caregivers/families, healthcare staff, and the wider healthcare system, using an established behaviour change model. To better understand the factors influencing engagement in providing or receiving care through a virtual ward, we applied the Capability, Opportunity, Motivation–Behaviour (COM-B) model [13], along with its extension, the Theoretical Domains Framework (TDF) [14], to guide our analysis of the enablers and barriers to virtual ward implementation.

The COM-B model is derived from existing theories of behavioural change [13]. The model proposes that interactions between capability (i.e. psychological and physical capacity to give, or receive, care on a virtual ward), opportunity (i.e. the external factors that enable or hinder the delivery, or receipt, of care on a virtual ward), and motivation (i.e. internal factors that drive someone’s desire to give or receive care on a virtual ward) lead to the performance of a particular behaviour (i.e. agreeing to give or receive care on a virtual ward). The TDF builds on these components of the COM-B model by identifying 14 specific domains that can influence behaviour, such as knowledge, skills, and social influences [14] (see Table 1).

**Table 1.** COM-B (Capability, Opportunity, and Motivation-Behaviour) factors and Theoretical Domains Framework (TDF) domains and definitions (adapted from Cane *et al*. [14])

COM-B components		TDF domains
Capability	Psychological	*Knowledge*—the knowledge required to deliver, or receive, care on a virtual ward. *Memory, attention, and decision processes*—the ability to retain information, and make decision related to delivering, or receiving, care on a virtual ward. *Behaviour regulation*—anything aimed at managing or changing objectively observed or measured actions within the context of delivering, or receiving, care on a virtual ward.
	Physical	*Skills*—the skills required to deliver, or receive, care on a virtual ward.
Opportunity	Social	*Social influence*—interpersonal processes that can cause individuals to change their thoughts and feelings about delivering, or receiving, care on a virtual ward.
	Physical	*Environmental context and resources*—any circumstance of a person’s situation or environment that impacts upon the ability to deliver, or receive, care on a virtual ward.
Motivation	Reflective	*Social/professional role and identity*—a coherent set of behaviours and displayed personal qualities of an individual expected when delivering or receiving care on a virtual ward. *Beliefs about capabilities*—a belief that someone is capable of delivering, or receiving, care on a virtual ward. *Optimism*—a belief that the delivery, or receipt, of care on a virtual ward can be carried out successfully. *Beliefs about consequences*—a belief that the delivery, or receipt, of care on a virtual ward can be achieved. *Intentions*—a conscious decision to deliver, or receive, care on a virtual ward. *Goals*—a desire to deliver, or receive, care on a virtual ward.
	Automatic	*Reinforcement*—a process that increases the probability of delivering, or receiving, care on a virtual ward by arranging a dependent relationship, or contingency, between the response and a given stimulus. *Emotions*—a complex reaction pattern, involving experiential, behavioural, and physiological elements that impact the willingness to deliver, or receive, care on a virtual ward.

The TDF is an elaboration of the COM-B model’s core components. The TDF breaks down these components by identifying 14 specific domains that can influence behaviour (see Table 1). This elaboration supports a more granular understanding of the factors that drive behaviour compared to those domains delineated in the COM-B model alone. The TDF serves as a practical, theory-based framework that supports the development of evidence-based implementation strategies [14]. The relevance and utility of the COM-B model and TDF have been demonstrated in previous qualitative systematic reviews examining barriers and facilitators to clinical behaviour change, such as the adoption of new practices in primary care [15] and adherence to prescribing guidelines [16].

## Methods

This review was conducted, and is reported, in accordance with the Preferred Reporting Items for Systematic Reviews and Meta-Analyses 2020 Statement [17]. A review protocol was registered on PROSPERO (Public Registry for Systematic Review Protocols in Health and Social Care) in March 2024 (Registration number: CRD42024519627).

### Search strategy

Electronic searches were conducted in Medline, EMBASE, CINAHL, PsycINFO, and Academic Search Complete in March 2024. A search strategy comprising Medical Subject Headings (MeSH) and free-text search terms was developed by the research team and supported by an examination of the search terms used in previously published systematic reviews on virtual wards [2, 3, 18]. It was further refined with the support of a research librarian from the University of Applied Sciences and Arts Northwestern Switzerland (FHNW) (for Medline search strategy, Supplementary Material S1). No publication date limitations were applied to the search.

### Study selection

#### Inclusion criteria

Studies were included if the main focus was on virtual wards. If not specifically stated, the following criteria were used to determine if an intervention qualified as a virtual ward: care is provided outside the healthcare facility (e.g. at home); involves a multidisciplinary team of at least two different specialties; involves the remote monitoring of vital signs (e.g. blood pressure, temperature); supports consultation with medical staff; and the primary goal is to facilitate early discharge and/or prevent inpatient admission into hospital by offering short-term transitional care. Further inclusion criteria required that the study use a qualitative design and report qualitative findings (mixed method studies were also included if qualitative data could be extracted); focus on barriers and facilitators to the successful implementation and sustainability of virtual wards; include only participants aged 18 years and older; and be published in English.

#### Exclusion criteria

Papers were excluded if: the focus was not on virtual wards; care was not provided outside of a hospital setting; a multidisciplinary team was not involved; remote monitoring of vital signs was not adopted; consultation with healthcare professionals was not supported; the virtual ward did not aim to facilitate discharge and/or prevent inpatient admission to hospital; qualitative data could not be extracted; barriers and facilitators to the successful implementation and sustainability were not examined; or the study was not published in English.

#### Screening

The returns from the database searches were exported to Endnote©. Duplicates were removed, and titles and abstracts were subsequently screened by S.C. Full-text screening was carried out by S.C. Any results with unclear suitability following full-text screening were reviewed and discussed with P.O.C (Paul O'Connor) until consensus was reached on its eligibility. A detailed record of inclusion/exclusion decisions was maintained on Microsoft Excel© throughout full-text screening (Supplementary Material S2).

### Data extraction

Data extraction was performed by two independent reviewers (S.C. and L.L.M.). The following descriptive information was extracted from each article: publication characteristics (title, name of authors, and year of publication); study characteristics (country in which the study was conducted, study design, data collection method, and recruitment context); participant characteristics (sample size, gender, age, illness/es, and profession); and characteristics of the virtual ward (assessment criteria, care team composition, monitoring devices, and consultation frequency). The two reviewers also extracted the following information on barriers and facilitators to the successful implementation and sustainability of virtual wards: all relevant quotes/verbatim comments from the participants in the original study and authors’ interpretations, whether or not supported by participants’ comments and quotes. The data extraction table was initially piloted on three studies. Any disagreement was resolved through discussion between S.C. and L.L.M.

### Quality assessment tool

The Quality Assessment with Diverse Studies (QuADS) tool [19] was used to evaluate the studies. The tool was selected based on its demonstrated content and face validity, strong inter-observer reliability, and its prior use in evaluating studies included in several virtual care reviews involving qualitative and mixed-methods research [20, 21], that are consistent with the types of studies included in our review. Two authors (S.C. and L.L.M.) completed the assessment together.

### Data synthesis

A deductive content analysis approach, as described by Elo and Kyngäs [22], was used to collate and organize the extracted data. Information on the barriers and enablers to the successful implementation and sustainability of virtual wards, as extracted from the papers, was categorized according to the domains of the TDF. The deductive content analysis followed a three stage process, as recommended when applying the TDF to qualitative data [23]: (i) preparation—becoming familiar with the data by repeatedly reading the extracted data; (ii) organizing—coding the data, grouping similar codes into categories, and mapping them to the TDF domains; and (iii) reporting—summarizing and presenting the findings. The coding and theme development were carried out by S.C. and then reviewed by P.O.C. and L.L.M. Any disagreements were resolved through discussion.

## Results

**Figure 1:**
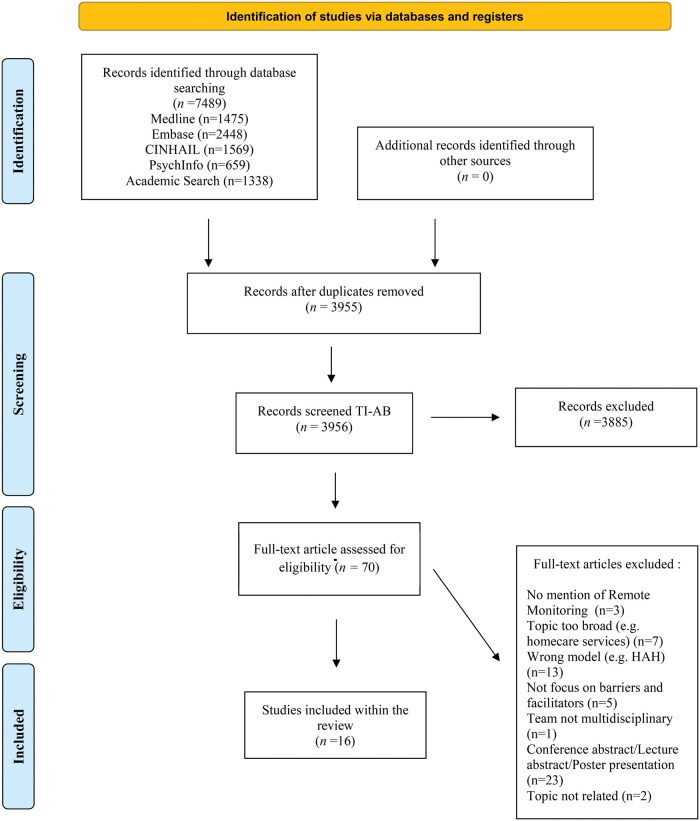
Flowchart of the study selection process following PRISMA 2020 guidelines

Database searches identified 7,489 articles. After reviewing the full text of 70 articles, a total of 16 studies met the inclusion criteria (Fig. 1). The participant group comprised 149 healthcare professionals—including obstetricians, midwives, nurse clinicians, general practitioners, physiotherapists, occupational therapists, pharmacists, residents, community matrons, and psychiatrists—; 146 patients with various conditions, such as high-risk pregnancies, COVID-19 (Coronavirus Disease 2019), diabetes, COPD (Chronic Obstructive Pulmonary Disease), heart failure, dementia, and psychiatric disorders; 33 caregivers (4 family members and 29 paid caregivers); and 16 administrative staff members.

Lee *et al*. conducted a survey with 60 healthcare providers as part of their mixed methods study. However, it was not clearly specified how many of these respondents contributed to the qualitative components, such as open-ended questions. Consequently, the precise number of participants providing qualitative data in this study remains unclear.

### Characteristics of included studies

Table 2 provides a summary of the characteristics of the included studies, with more details presented in Supplementary Material S3.

**Table 2.** Summary of included studies

	Number	%
Country of study		
Canada	2	12.5
UK	5	31.3
Australia	3	18.8
Norway	2	12.5
Denmark	1	6.3
Israel	1	6.3
Singapore	2	6.3
Participants		
Healthcare professionals	14	87.5
Patients	9	56.3
Carers	6	37.5
Administrative staff	5	31.3
Study design		
Qualitative	10	62.5
Mixed methods	6	37.5
Methodology		
Interview	12	75
Focus group	3	18.8
Questionnaire	3	18.8

### Quality assessment

The mean QuADS score was 22.3 (range 0 - 32). The quality scores of the majority of studies were reduced due to a lack of rationale for the choice of data collection tools or selected analytical methods. Additionally, many studies failed to demonstrate the involvement of research stakeholders in the design or conduct of the research.

### Synthesis of findings

Table 3 provides a summary of how the barriers and enablers were coded using the TDF (for more detail see Supplementary Material S4a and S4b ). To aid clarity, the findings are discussed according to the capability, opportunity, and motivation components of the COM-B model from which the TDF domains were derived.

**Table 3.** Summary of the number of times the TDF domains were used to code the barriers and enablers in virtual wards identified in the included papers

TDF domains	Number	%	Paper references
Capability			
Knowledge	16	100	[9–12, 24–35]
Skills	15	93.8	[9–12, 24–34]
Behavioural regulation	2	12.5	[9, 33]
Memory, attention, and decision processes	8	50	[9, 10, 26–28, 32, 33, 35]
Opportunity			
Environmental context and resources	16	100	[9–12, 24–35]
Social influences	14	87.5	[9–11, 24–31, 33–35]
Motivation			
Social/Professional Role and Identity	11	68.8	[9–12, 25, 27–32, 34, 35]
Emotion	11	68.8	[9, 10, 12, 24, 27, 28, 30, 32–35]
Beliefs about consequences	8	50	[9, 12, 24, 28–30, 33, 35]
Reinforcement	9	56.3	[9, 10, 12, 24, 26, 30, 33–35]
Beliefs about capabilities	6	37.5	[9–11, 24, 29, 30]
Goals	5	31.3	[9, 11, 24, 25, 31]
Optimism	3	18.8	[9, 27, 30]
Intentions	11	68.8	[9–11, 24, 28–35]

#### Capability

The Capability component of the COM-B model refers to an individual’s knowledge, skills, cognitive abilities, and self-regulatory capacity to deliver or receive care within a virtual ward setting. All four capability domain elements from the TDF were identified in the included papers, with the knowledge and skills domains most frequently identified (see Tables 1 and 3).

Patients and family members highlighted the risks and challenges of engaging with virtual ward care, particularly stemming from limited knowledge of the system, their medical conditions, or insufficient language skills—affecting both patients and caregivers alike. One participant remarked: ‘We know how to use [the vital sign monitoring] because we [are] only 50+ [years old], and we understand English. For old people, I think it’s not suitable… they don’t know English. They don’t know how to measure their blood pressure, [and] oximeter…’ ([35], p.4).

The need for technologically astute, knowledgeable, and specialized healthcare professionals was identified as a key facilitator of virtual wards. Participants in the included studies frequently emphasized the importance of experienced and skilled providers who communicate effectively and deliver high-quality care. A healthcare professional from one of the included studies noted that: ‘when we have competence and skills with using different assessment tools, I think we contribute to faster treatment of patients with exacerbations and thus reduce the severity of complications’ ([11], p. 681). Some providers expressed concerns about assessment accuracy and the ability to build rapport since virtual assessments require a different skill set. For example, providers must often rely on verbal cues and ask more detailed questions due to limitations in video quality: ‘see, touch, listen is key of practice training of nurses and nurse assistants… You need specific competence development focusing on how to be a remote nurse’ ([9], p. 6).

Several studies identified issues for the healthcare system. The need for clear, standardized eligibility criteria and protocols for admission, care delivery, and discharge is essential to support the rapid and sustainable implementation of virtual wards. For example, one participant from an included study remarked: ‘they created a shared drive which the majority of our information went into; introduction packages that we sent to patients. The policy itself is on Prompt [hospital intranet], templates we used when speaking with patients, so that it's consistent … was emailed … and was on the [shared] drive so you could access it yourself, and as they got updated, they emailed all of us so that way if there were any changes we knew straight away’ ([10], p. 7).

#### Opportunity

The Opportunity component of the COM-B model focuses on the external factors that influence the delivery or receipt of care within a virtual ward, such as available resources or contextual elements like social influences. Both TDF domains associated with this component were reflected in the included studies (see Tables 1 and 3).

Patients highlighted challenges with the monitoring of vital signs (e.g. instability of transmission of readings). One participant remarked: ‘once I go shower, the patch will not stick again… and you sweat so much, it will come out’ ([34], p. 696). To help mitigate some of these challenges, several papers have pointed to the importance of a strong support system, often involving next of kin, paid caregivers, or acquaintances. One healthcare provider explained: ‘she [the domestic helper] sleeps just outside his [the patient’s] room…he would know how to call if he needs help…she’s [the helper’s] is very responsive…’ ([34], p. 695). The support system could also involve medical professionals. For example, a patient in one of the included studies stated that: ‘the nurse was very good, can’t praise her really high enough. She was a friendly voice to speak to’ ([33], p. 2398).

For healthcare providers, nine studies [9, 26–29, 31–34] identified patients’ limited access to internet, technical difficulties like connectivity problems, outdated devices, and unfamiliarity with digital tools as significant barriers to delivering virtual care: ‘I did the first video conference for one of the patients, I can connect somehow, but it’s so hard, you know?’ ([29], p. 7). Providers also emphasized the importance of mutual trust and respect among colleagues from different professions: ‘Initially I was very hesitant to work here because I’ve worked in ED for almost 10 years and I hate change but because ED is not safe for me at the moment, I was offered… I mean they wanted me to get redeployed in this job and initially I thought oh my god, I don’t know I can do it. From day one they have been welcoming and I didn’t get intimidated at all because my suggestions were always welcome, they would always listen and stuff so yeah I’m just… I’m thankful that I have been redeployed here’ ([10], p. 10).

A key implication for the healthcare system is that the availability of adequate resources—such as equipment, staffing, and funding—is essential to effectively support the implementation of virtual wards. For example, it was noted in one study: ‘when it comes to human resources, the resources for all the health professions are limited’ ([12], p. 4), and ‘financial considerations threaten the initiative’ ([12], p. 4).

#### Motivation

The Motivation component of the COM-B model encompasses internal factors that influence an individual’s willingness to deliver or receive care on a virtual ward, including motivation, beliefs about one’s capabilities, optimism, and more. All eight TDF domains associated with the motivation component were reflected in the included studies (see Tables 1 and 3).

For patients and family members, adapting to virtual ward care often required taking on new roles, with caregivers shouldering particularly significant responsibilities. For example, a participant described this experience as overwhelming: ‘I found, to start with I found the text messages useful but the longer they went on the more irritating. I was, I felt like I was chained to the phone and you know and to my equipment. So three times a day is, I know that’s necessary to start with but I just felt that maybe twice a day after that might have been better’ ([33], p. 2399). Caregivers, in particular, were expected to function as first responders despite lacking formal healthcare training. One healthcare provider stated: ‘Family members become part of the treatment staff’ ([12], p. 3). Despite these concerns, both patients and caregivers found the home environment less stressful and more private compared to the hospital and praised the sense of comfort and security that family members, and especially healthcare professionals, were able to create: ‘…comfort, it is intangible right. You can’t put dollars and cents into it…ability to sleep better right, you are close to the things that you are used to…watch TV…go onto the computer. Where else in the hospital, you can’t do all these’ ([35], p. 693).

For healthcare providers, it was important to have strong executive engagement and proactive leadership, especially when it came to coordinating and sharing information among the healthcare professionals: ‘the nurse who leads the CVW [Community Virtual ward] always asks us what to report to the other professionals’ ([11], p. 682).

For the healthcare system, the positive reception of virtual wards was seen as a key facilitator of broader acceptance, particularly because stakeholders such as administrators, clinicians, and caregivers were more likely to support the model when they saw their peers engaging with it successfully. However, not all parts of the healthcare system were fully aligned behind the concept. Some healthcare providers emphasized the need for a cultural shift, with one stating: ‘we need to build a new practice philosophy’ ([11], p. 4).

## Discussion

### Statement of principal findings

Our review used the TDF [14], an elaboration of the COM-B model [13], to classify the barriers and facilitators to virtual wards. Within the Capability domain, the key TDF areas identified were ‘Knowledge’ and ‘Skills’. Many participants highlighted that patients and caregivers face significant challenges in virtual wards due to limited technological knowledge, medical literacy, or language barriers, and emphasized the need for mandatory training to address these issues. Our review also highlighted significant facilitators within the Opportunity component, specifically related to the TDF domains of ‘Environmental context and resources’—notably the need for appropriate resources—and ‘Social influence’—the presence of a strong support system. Within the Motivation domain, facilitators were identified in the TDF areas of ‘Social/professional role and identity’, mainly support from main stakeholders, ‘Emotions’, the ability of healthcare providers to create a reassuring environment, and ‘Intentions’, involving disciplined individuals, indicating the need for targeted efforts to address these factors. The following paragraphs explore these issues in greater depth, incorporating insights from related health services research—such as co-design and simulation training—to propose practical, stakeholder-informed strategies for addressing capability gaps within the virtual ward context.

A lack of knowledge and skills related to remote care undermines the viability of virtual wards and poses a risk to patient safety and the quality of care. These include conducting online consultations and performing tasks such as measuring body temperature or blood pressure remotely. A potential approach to minimize knowledge and skill barriers to virtual wards is to co-design virtual wards with end users [36]. Co-design refers to ‘the creativity of designers and people not trained in design, working together in the design development process’ ([36], p. 6). Instances of the use of co-design to develop virtual care models are beginning to appear in the literature. For example, Jackson *et al*. [37] outline a protocol to develop a virtual hospital in which co-design focus groups with healthcare professionals, patients, and carers make recommendations on how to foster a good patient experience. This collaborative approach helps bridge knowledge and skill gaps by fostering a shared understanding and ensuring that technology and processes are tailored to the abilities and needs of both patients and healthcare professionals to support the delivery of safe and effective care [37]. Therefore, researchers and policymakers should consider how to incorporate input from all stakeholders involved in virtual wards to design technologies that effectively support the delivery and receipt of care from the perspectives of patients, caregivers, and healthcare providers.

### Interpretation within the context of the wider literature

In the absence of sufficient resources, there is the risk that virtual wards could add to the workload of already busy healthcare professionals and negatively impact their mental health, the quality of care, and patient safety [9, 28]. Using a co-design approach, that also includes healthcare managers, will help identify the specific resources required, and how they can be accessed and ensure that limited resources are being used where they are most needed [36]. Another critical aspect to consider is the role of trust and support. As patients are being cared for virtually, there is the potential for them to feel isolated and/or forgotten by healthcare professionals [33]. Peer support is a process through which people with shared common experiences come together as equals to give and receive help based on the knowledge derived from their similar journeys [38]. We suggest developing peer support programs for patients in virtual wards to enhance their care experience and outcomes. This would be beneficial in reducing the feeling of isolation by providing a network of others in a similar situation.

Increased responsibilities and a lack of preparedness to give and receive care on a virtual ward can lead to feelings of loss of control among healthcare professionals,disengagement from patients, and negatively impact patient safety and quality of care [9, 30, 35]. Simulation-based education could serve as an effective training method benefiting not only healthcare professionals but also patients and carers. This approach can enhance their confidence by ensuring they are well-prepared to perform the necessary tasks expected of them [39]. There is substantial evidence supporting the effectiveness of simulation-based education in training healthcare professionals [40, 41]. However, simulations could also be used to allow patients and carers to practice the tasks they will be expected to perform in a safe learning environment [42]. For example, simulation-based training for patients with kidney disease on performing home haemodialysis has been linked to a reduced need for home visits from healthcare professionals [43]. This suggests that this approach can enhance patient independence and boost their confidence in managing their own care.

### Implications for policy, practice, and research

Technological improvements are set to revolutionize virtual wards, with innovations such as smart home systems providing new avenues for care delivery [44]. However, to fully realize the potential of virtual wards, the implementation challenges associated with this model of care must be clearly identified and effectively addressed. A common theme in the quality improvement literature is that achieving meaningful change in healthcare is inherently challenging [45]. Therefore, to effectively translate research into practice, researchers should apply principles of behavioural science change to design virtual wards that support a desired change in behaviour and enable both patients and healthcare providers to engage with and adopt virtual ward models. Policy makers must consider the implementation of virtual wards, which can be supported through the application of suitable behavioural change models [13]. Our review has identified the importance of addressing the issues of capability (e.g. the need for enhanced knowledge, awareness, and specialized training), opportunity (e.g. the potential to harness social influence), and motivation (e.g. the importance of engaged and proactive leadership) that should be addressed when designing a virtual ward.

### Limitations

There are a number of limitations to this systematic review that should be acknowledged. First, although two reviewers conducted the data extraction independently, the findings represent the reviewers’ interpretations of the findings from the papers. Second, we only included papers related to virtual wards, and did not consider the barriers and facilitators to other types of virtual healthcare models (e.g. privacy and security breaches, large variations in regulations, etc.). Finally, only qualitative data were considered in this systematic review, which can lead to concerns about researcher bias and generalizability and may have resulted in the omission of some useful quantitative data.

## Conclusion

Virtual wards offer a promising solution to alleviate the hospital capacity crisis, and support the delivery of safe, high-quality care within patients’ home environments. Moreover, emerging technologies will increase the patient population that could potentially benefit from care on virtual wards. However, to fully realize the potential of virtual wards, it is important to understand the barriers and facilitators affecting patients, carers, and healthcare providers. This review enhances understanding of key issues, enabling the development of targeted strategies and interventions to support both the delivery and receipt of safe quality care on virtual wards.

## Supplementary Material

mzaf065_Supplementary_Data

## Data Availability

The data supporting this systematic review are available in the Supplementary Material of this article. All relevant data, including study details and thematic coding, are provided there.
