# Supplementary material for: Barriers and facilitators to the use of virtual wards: a systematic review of the qualitative evidence
Source: Int J Qual Health Care. 2025 Jul 18;37(3):mzaf065. doi: 10.1093/intqhc/mzaf065 (PMC12342918; doi:10.1093/intqhc/mzaf065)
Supplement: mzaf065_Supplementary_Data [file mzaf065_supplementary_data.zip › INTHQC-2025-02-0056.R2_FINAL SEARCH STRATEGY (Supplementary Material 1).pdf]

Supplementary Material 1: Summary of Medline OVID Search Strategy

---

1. (Virtual adj3 Care).ti,ab
2. (Virtual adj2 care adj2 servic*).ti,ab
3. (Remote adj3 Ward*).ti,ab
4. (Virtual adj3 Ward*).ti,ab
5. (Remote adj2 Home adj2 Monitor*).ti,ab
6. (Virtual adj2 Home adj2 Monitor*).ti,ab
7. (Hospital adj3 Home).ti,ab
8. 1-7/OR
9. Hurdle*.ti,ab
10. Obstruct*.ti,ab
11. Discourag*.ti,ab
12. Facilitator*.ti,ab
13. Challeng*.ti,ab
14. Enabl*.ti,ab
15. Barrier*.ti,ab
16. Imped*.ti,ab
17. Concern*.ti,ab
18. Caus*.ti,ab
19. Advantag*.ti,ab
20. Disadvantag*.ti,ab
21. Burden*.ti,ab
22. Benefi*.ti,ab
23. Positiv*.ti,ab
24. Negativ*.ti,ab
25. Issue*.ti,ab
26. Problem*.ti,ab
27. 9-26/OR

28. Exp Qualitative Research/
29. Exp Grounded Theory/
30. Exp Interview/
31. Exp Focus group/
32. Qualitative.ti,ab
33. (Grounded adj1 Theor*).ti,ab
34. Interview*.ti,ab
35. (Focus adj1 Group*).ti,ab
36. Ethnograph*.ti,ab
37. (Mixed adj1 Method*).ti,ab
38. Mixedmethod*.ti,ab
39. (Multi* adj1 Method*).ti,ab
40. Multimethod*.ti,ab
41. Phenomenon*.ti,ab
42. Narrative*.ti,ab
43. (Content adj1 Analys*).ti,ab
44. (Them* adj1 Analys*).ti,ab
45. Discourse.ti,ab
46. (Document adj1 Analys*).ti,ab
47. (Textual adj1 Analys*).ti,ab
48. Fieldwork.ti,ab
49. 28-48/OR
50. 8 AND 26 AND 49

Note Exp=explode, adj=adjacency operator, ti=title, ab=abstract
