# Supplementary material for: Barriers and facilitators to the use of virtual wards: a systematic review of the qualitative evidence
Source: Int J Qual Health Care. 2025 Jul 18;37(3):mzaf065. doi: 10.1093/intqhc/mzaf065 (PMC12342918; doi:10.1093/intqhc/mzaf065)
Supplement: mzaf065_Supplementary_Data [file mzaf065_supplementary_data.zip › INTQHC-2025-02-0056.R2_ TDF table single studies (Supplementary marterial 4b).docx]

**Supplementary Material 4b: Data extraction barriers/facilitators coding TDF Model**

| **First author (year)** | **TDF domain** | **N of TDF domains** |
| --- | --- | --- |
| **Asabo (2024)** | **COM-B: Capability**  **TDF Model: Skills**  **Barriers**  **Assessment accuracy**  Remote follow up limits such possibilities to use the clinical gaze and assess symptom.  touching pregnant women's bodies and abdomens, was viewed as crucial for assessing stability and changes in condition  Women expressed that it can be difficult to assess what is normal and what is an indication for exacerbation of risk.  *“We are the ones to assess, and if I work with that group, I'd like to see the patient. If she's bleeding, I'd like to look at the pad to see how much it is. “Is it a drop?” Or is it “a real bleeding?” Because this is exactly what women fail to assess” (Midwives)*  **Communication**  The obstetricians suggested it could be challenging to communicate risk and to instruct HOME patients during acute situations such that they would take the situation seriously but not be overly frightened  *“So it's also an educational challenge for us, to be able to inform them sufficiently, without scaring the water out of them. Because in theory they can actually die”(Obstetricians)*  **Facilitators**  **Competence/experience**  Available and competent midwives and Obstetricians are crucial to empower and enable an active patient role, particularly in acute situations. Availability of professionals was regarded as crucial to feel safe.  **TDF Model: Knowledge**  **Facilitators**  **Patient and family knowledge**  In addition, they emphasized the necessity to assess the women's [..], health literacy  In addition, they emphasized the necessity to assess the women's [..] as well as level of Norwegian language/communicative skills before offering HBTM  *“The training should not just be instrumental—“You put it on like this, and then you press there”—without you getting an understanding of what you have measured, because I think that's important” (Bereaved)*  **Educational resources**  All groups emphasized the importance of thorough, tailored information provision and training at the hospital for the women to be able to perform the routines of HBTM.  **Eligibility Criteria/Procedural Guidelines**  Midwives and obstetricians highlighted the importance of unambiguous and standardized criteria for patient eligibility.  In relation to this aspect, they emphasized the importance of clear guidelines and procedures, when critical and acute situations occur  They underlined that it is crucial to know when women need to be transferred to hospital and, hence, not leave it up to the women to assess the necessary actions  The midwives pointed out that the inclusion criteria for HBTM may not necessarily fit clinical reality, as women with high-risk pregnancies often have highly complex risk profiles (eg pPROM with fetal breech position that requires proximity to a delivery service).  Some midwives and obstetricians argued that being monitored at home may be challenging for some groups, particularly women with small children or other family care obligations  **Facilitators**  **Eligibility Criteria/Procedural Guidelines**  Obstetricians and midwives stressed the need for individual assessments of patient eligibility for HBTM, taking into consideration the patient's life/family situation, distance to hospital, as well as the resources and supporting networks of each woman  *“I understand very well that it [remote monitoring] helps women who have high blood pressure, who have a tendency to preeclampsia, because these things will be picked up. And that's why I think they just have to select the right patient group for that” (Bereaved)*  **COM-B: Opportunity**  **TDF Model: Environmental context and resources**  **Barriers**  **Resources**  Staff availability was also seen as dependent on hospital management. High work pressure and staff shortages would mean that HOME affiliated midwives in the ward and occasionally be forced to deprioritize HOME patients  However, external dimensions such as a highly pressured specialist health care, staff shortages and the task of integrating and coordinating flexible and personalized services may present additional challenges in introducing an HBTM model  Participants expressed concern regarding contact availability outside office hours, i.e., phoning/contacting the Department of Obstetrics alongside all other pregnant women  **Facilitators**  **Resources**  *“It's sort of convenient for us to have the patients hospitalized ensuring that everyone is monitored all the time. But it [an emerging acute situation] may just as well happen while you are admitted to the hospital as when you are at home. But, I think you are getting a much more efficient utilization of health professionals. And if the availability [of healthcare professionals] then for the patients who are at home is good, then I think we'll manage to maintain the patients' sense of safety” (Obstetrician)*  **TDF Model: Social Influences**  **Barriers**  **Social Support**  The women were primarily concerned whether home-monitored women risked being left alone without sufficient support and care  **Communication**  The lack of face-to-face communication with women makes the clinical follow up more challenging.  **Facilitators**  **Support**  Not being left alone with medical equipment they then must handle  *“[It's important] that the patient is not left with a feeling of responsibility for their own treatment. As long as you have someone you can talk to, who you physically talk to who can explain and reassure you, then you feel that someone else has responsibility” (Hospitalized)*  **Communication**  Women with high-risk pregnancies emphasized that experiencing good communication with midwives and obstetricians was crucial to maintaining a sense of safety According to the obstetricians, discharging women with at-risk pregnancies from the hospital implies a substantial cultural change in how midwives and obstetricians work, more explicitly based on building trust [..]  **COM-B Model: Motivation**  **TDF Model: Emotions**  **Barriers**  **Anxiety/stress**  It was challenging for women who had experienced stillbirth to handle the risk of a new pregnancy along with limitations of treatment and follow up, despite their awareness that pregnancies are never 100% free of risk. In subsequent pregnancies, they had experienced anxiety and therefore a substantial need for being in control.  **Facilitators**  **Comfort**  According to the obstetricians, for specific patient groups (eg hypertension), the home setting may be a better place for follow up because it is more comfortable and less stressful than the hospital and may have positive effects on BP  *“[It's important] that you do not feel forgotten, because some may feel that if they are sent home and hear, “Yes, here you are, here are some devices you are to use” and then you are out of sight and out of mind! It's just that you sort of get the feeling that you are being taken care of then, even if you are staying at home.” (Hospitalized, Interview 1)*  **TDF Model: Reinforcement**  **Facilitators**  **Feedback**  Women emphasized the importance of receiving some sort of confirmation that their data had been received and assessed by midwives/obstetricians. Short phone calls a few times a week with a dedicated HOME midwife and available competent/familiar midwives and obstetricians for ad hoc inquiries/consultations were seen as crucial. Hence, predictable and reliable follow-up contact routines were highly valued  Timely (re)actions to measurements  **TDF Model: Goals**  **Facilitator**  **Health-seeking behaviour**  In addition, they emphasized the necessity to assess the women's [..] health-seeking behavior  **TDF Model: Intentions**  **Barriers**  **Discipline**  One patient described herself as lazy by nature and therefore a little sceptical when introduced to the HaH concept.  **Facilitators**  **Discipline**  It was emphasized that such monitoring must be voluntary and cannot be viewed as a substitute to current hospital-based services simply to save costs  In addition, they emphasized the necessity to assess the women's expected compliance with HBTM [..]  *“You have to be extremely determined. Because you, you actually have to demand quite a lot yourself. At least in a situation like the one we have been through, it's not someone who just gives you things… you actually have to stand up and demand that: “I need this!” And like, “What do you need?” No one is asking you that. You kind of have to demand a bit yourself then […] You have to fight for yourself in the system!” (Bereaved)*  **TDF Model: Beliefs about consequences**  **Barriers**  **Expected loss of control**  They were worried that reception staff would not properly understand/take seriously the high clinical risks of HOME patients.  Based on their clinical experience, they know that during a high-risk pregnancy, some women struggle with understanding (and caring for) their pregnancy complications, emotions, and needs  They questioned whether women at home were able to communicate emergencies.  The current study also indicates that if women with a risk pregnancy lack available and competent professional support, both women monitored at home and midwives and obstetricians are likely to experience loss of control and safety  *“What if suddenly they do not, if something dramatic happens in the maternity ward? Does the midwife still have time to check the results? So that's what I'm a bit concerned about. “How prioritized are you when you are sitting at home vs those who are lying in bed and giving birth in the hospital?” (Bereaved)*  *“[We have] control in a different way. Because when the patient is at home, you give the responsibility to them, then it's the patient who has responsibility. And that's a bit hard to give up that responsibility” (Obstetrician)*  **Avoiding negative consequences**  Some women had experienced gatekeeping from reception staff, and their awareness of stress and work pressure in the ward made them hesitant to make contact, fearing that they would not be taken seriously or would even be viewed as hysterical  *“And I think it's really important to think about how women are asked the question about whether they'd like to [have remote monitoring]. Because in my experience, there are a lot of people who are afraid of taking up too much space here, or disturbing already very busy people who work here. Yeah, so they do not just say yes to be polite” (Hospitalized, Interviewee 5)*  *“The CTG measurements at home, then. That is, if you suddenly get a high pulse on the child, or something urgent happens at home, what, how to deal with it? Because I think that if you are going to run CTGs in the first place, it's because there's a need to monitor the child, and then you also need to be able to act quickly if something is abnormal. So that's the part I'm most concerned about, how they will deal with that.”(Hospitalized, Interviewee 3)*  **TDF Model: Beliefs about capabilities**  **Barriers**  **Empowerment**  *“Some patients you have no problem sending home because you think, “She understands when something is in the offing and takes action, and she knows where to call.” But then there are a few who you thought would know: “Gee, but why did you not call a week ago?” In a way, you get the feeling that they do not quite manage to take care of themselves then or understand the signals and react in an adequate way. And it's clear that then it kind of sticks, or at least you have a lower threshold for hospitalization and follow them up more closely” (Obstetricians)*  **Perceived behavioural control**  *“[We have] control in a different way. Because when the patient is at home, you give the responsibility to them, then it's the patient who has responsibility. And that's a bit hard to give up that responsibility” (Obstetrician)*  **Facilitators**  **Perceived behavioural control**  *“2:Yes, but then you should be confident in yourself as a midwife, right? The patient will feel safe if you [the midwife] give clear messages, and are very certain about what you say. And, not like, “yeah, maybe” or “I'm not quite sure,” because then I believe... 1: Yeah, so you have to select midwives too [laughs]. 2: Yeah, they should not be a fresh educated midwife. [...] 3: Yeah, but it must be important that the women who will be involved and are to be monitored at home, that they feel self-efficacy and will cope. That must be the most important thin” (Midwives)*  *“What they (the nurses) have worried most about, I think, is if the patients are capable of monitoring properly”*  *“After all, I'll feel a great need to spend some time with that patient and sort of get an overview of resources. Like, “What can they do? To what extent are they compliant enough to take responsibility for…?” And, “Do we know if they report to us?”(Midwives)* | **10** |
| **Cerdan de Las Heras (2023)** | **COM-B Model: Capability**  **TDF Model: Behavioral regulation**  **Barriers**  **Self-monitoring**  Obtaining vitals at night was an overwhelming challenge  **Facilitators**  **Self-monitoring**  Beyond measuring, patients experienced how they kept an eye on time and schedules and prepared to be relaxed and thus ready for blood pressure measuring  **TDF Model: Memory, attention and decision process**  **Barriers**  **Poor health/tiredness**  *“If I think it is a problem, if I need to wake up at night to measure those values and I feel really exhausted and unconcentrated, then I prefer to be at the hospital”(Informant ID 14/1902)*  *The only thing I was upset about was the night I should do the measuring; I hadn’t slept for two nights. [ . . . ] and I have had a sleeping pill 112 -2 h before (Informant ID 11/2147)*  **Cognitive capacities**  Simultaneously taking care of both the usual and virtual patients was perceived as confusing and perceived inappropriate for the future  *“You need to respond to two different groups of patients, when attending shift”(Informant ID 8/1189)*  *“With a patient in front of you, I think, it will be difficult to prioritise a remote patient, one that you can’t see, instead of the one in front of you who may be in pain or something else. There are some practical issues, I see Ole here, but I can’t see Maren who is alarming my phone”(Informant ID 5/1410)*  *It just means that a nurse taking care of patients, needs to drop everything to address an alarm within five minutes; a clinical situation that is not critical, professionally critical [...] I am concerned that we will then use resources for unnecessary tasks (Informant ID 13/1788, 1802)*  *It is a question about how far you are in your disease trajectory, and how cognitive well bright you feel and so. But, there are tasks that you need to remember. But, difficult, no it is not difficult, for sure!(Informant ID 9/1137)*  **Facilitators**  **Cognitive capacities**  clinicians imagined their attention being more undisturbed when the time was scheduled and the room was emptied for other clinicians and patients  **TDF Model: Skills**  **Barriers**  **Assessment accuracy**  *“The patients left will be more complex and require a lot of care ’See, touch, listen‘ is key of practice training of nurses and nurse assistants [ . . . ] You need specific competence development focusing on how to be a remote nurse”(Informant ID 4/472,484)*  *“I need to get used to that they (the patients) are not just on the other side of the door, that I cannot just . . . [ . . . ] When washing a patient or mobilise one, or whatever you do, you notice [ . . . ] there are so many things that you talk about and notice yourself, and. You will miss some things. [ . . . ] You do not get information easily”(Information ID 2/277)*  **Facilitators**  **Communication**  It bothered informants how to build a professional relationship with virtual patients  First, clinicians were free from personal protective equipment, allowing patients to read body signals and to better hear. Especially for patients hospitalised for extraordinarily long periods, dialogue with a clinician not fully equipped was perceived crucial.  *“Without being aware of it, we continuously use body language, when we are talking right now, when we are talking using FaceTime, we do see the other person’s face and decode how our words have an impact on the other person. That is not possible when you are all wrapped in the personal protective equipment. You cannot decode anything at all. You cannot use your normal decoding techniques to actually understand the other person and read how the other person receives your information. And we are facilitating that”(Information ID12/2297)*  *“If it is a COVID-patient, you go there totally covered up in your personal protective equipment, the patient might not know if it is the doctor or the nurse. Who is visiting me? Who is asking me all these questions? Then I do believe, with respect to communication, that it can be an advantage, that you are not covered with a mask, glasses and other personal protective equipment, that you can see the person. There is also a concern on patients with hearing loss, who need to lip read, but that is not possible when we are wearing all the personal protective equipment”(Informant ID 3/74)*  **Training/learning**  Both nurses and doctors were concerned about their own and future colleagues’ skills for examination and treatment at distance. The introduction and training program mainly covered technical issues. Some felt left in limbo—in doubt how to accommodate patients at distance. It was recurrently mentioned how appropriateness of nurse and doctor professional skills cannot be taken for granted and include several competences beyond the digital  If appropriately introduced, treatment based on self-monitoring and remote communication was perceived acceptable for the patients  Several reassured themselves that practicing would show and also expose a likely need for focused training. Specialisation for virtual nursing, continuous medical education, and revision of medical curricula were proposed  *“I think, if I have had a training period long enough to convince me that from now, with no doubt, I can send the right values; then I would feel completely safe with it”*  *The more experienced you become, the more relaxed you will be in your professional position. But I am sure, that when we start to include the first patients for HaH, then, staff will perceive the situation as very insecure” (Informant ID 14/1752)*  **TDF Model: Knowledge**  **Barriers**  **Patient and family knowledge**  Elderly and others with low digital competence were to be excluded  **Knowledgeable staff**  *“Challenges with education of the staff—to make them feel safe in the process, and how are all the workflows, because it is an all-new way of thinking. To have a virtual work environment instead of a physical”(Informant ID 5/1370)*  *“They (the oximeters) are made for the nurses to read. To say, the number being 99 at my first monitoring looked like 66 to me (laughing). But, then I quickly saw, that, of course [ . . . ] I just saw it upside down.”(Informant ID 6/570)*  **Eligibility Criteria/Procedural Guidelines**  For safety reasons, exclusion criteria could be so restrictive that the patient volume becomes too low to reach break-even for costs and professional comfortability and competences  Owing to mental unawareness and physical challenges, not all patients may be ready for the concept, several patients explained. It was highly underlined that the model was ‘for the right patients—not all’  **Facilitators**  **Patient and family knowledge**  The need for matching expectations regarding patient responsibility and tasks was highlighted  Being at home will furthermore make it easier for relatives to be updated on the disease course and severity  *And then I do also believe [ . . . ] maybe it is easier for the relatives because they can follow the disease trajectory and do not have to call the department all the time when they know we are busy.(Informant ID 5/1312)*  **Knowledgeable staff**  *“If healthcare professionals, both doctors and nurses, are educated like 50 years ago, can it then be expected, that they can handle a digital patient? [...] We educate people to make patient evaluation with stethoscope and manual examinations, what do we then do, when the patients suddenly are presented on a screen instead of the physical meeting?”(Informant ID 3/150)*  **Eligibility Criteria/Procedural Guidelines**  Several patient groups were suggested for future models, such as chronic patients who could restabilise within a short time, dehydrated elderly, patients with COPD exacerbation, or patients in long-term intravenous antibiotic treatment. Future patients need to be stable with only low risk of acute treatment needs, but clearly in need of monitoring and professional contact 24/7, i.e., in need of hospitalisation if this model did not exist. Additionally, reverse models with special out-going facilities were considered  *“It is a considerable job. And there are many (COVID patients that may deteriorate fast) [ . . . ] All these flow diagrams made in case we do not reach the patient, what then to do? [ . . . ] I think a nice safety net has been established”(Informant ID 2/39)*  *“We have to change mentality among staff and patients, so it gets an integrated part of the definition of being admitted to hospital, that this actually also can happen at home.” (Informant ID 5/1568)*  *“Owing to mental unawareness and physical challenges, not all patients may be ready for the concept, several patients explained. It was highly underlined that the model was ‘for the right patients—not all’(Informant ID (1/441)*  **COM-B: Opportunity**  **TDF Model: Environmental context and resources**  **Barriers**  **Resources**  However, others underlined the unknown need for resources. [..]were afraid of hidden overcrowding.  *“It just means that a nurse taking care of patients, needs to drop everything to address an alarm within five minutes; a clinical situation that is not critical, professionally critical [...] I am concerned that we will then use resources for unnecessary tasks”(Informant ID 13/1788, 1802)*  **Technical/technology use**  A high number of teething troubles with communication equipment bothered everyone, and failures affected their professional self-respect negatively.  The poor integration of the project platform and the electronic health record was time-consuming. Several still did not consider the HaH concept and equipment ready or valuable. To diminish patient failures due to data feeding, one argued for Bluetooth systems  Running for alarms, failures of technology, and systematic monitoring in line with algorithms but untailored the individual patient were referred to as meaningless and upsetting job satisfaction  Perceived technical problems with the digitalisation of communication included adaption on older mobiles, download of the secured app, missing internet connection, reverb on a noisy line, and an unaccustomed low resolution of the video  Using the phone for work made it difficult and close to unacceptable for a few to break this practice to make the app work  *“When transitioning a patient for HaH, you are dependent on—that technology works”* (Informant ID 8/1095)  *When it works, and there are no more teething problems, I think, then they (the nurses) see it. Just now, I do not think they find it positive at all (Informant ID/2/257)*  **Standardized protocols**  Both nurses and doctors worried about norm agreements  At the same time, fixed appointments were by nurses experienced as saving them small breathers  **Facilitators**  **Resources**  *“My hope is and has been throughout the project, that some clinicians will be dedicated to care for the virtual patients, and only take care of them”(Informant ID 5/1406)*  **Technical/technology use**  Generally, the patients concluded that the devices were easy to understand and use, ‘almost faulttolerant’  **TDF Model: Social influences**  **Barriers**  **Communication**  Worries mainly concerned patients never meeting face to face with health professionals, but also, as a doctor explained, how to support continuity of care across transmission to HaH.  **Facilitators**  **Support**  Being contacted in case of exacerbation gave patients the feeling of individual interest and empathy from the clinicians.  It was foreseen that older patients may need support for HaH activities at home, and as explained by one who had called her mother to stay, severely diseased people may require help to manage the oxygen supply, shopping, etc.  *(talking about having the oxygen cylinder at home) “I notice how I become, and then I ask my mother to go out and turn up, because I’m in trouble”(Informant ID 11/2493)*  *“Yes, you do (become dependent on other people). But, fortunately, I have lots of nice neighbours who have all said to me ‘in case you need groceries or something else, just call”(Informant ID 11/2612)*  **Communication**  *Without being aware of it, we continuously use body language, when we are talking right now, when we are talking using “FaceTime, we do see the other person’s face and decode how our words have an impact on the other person. That is not possible when you are all wrapped in the personal protective equipment. You cannot decode anything at all. You cannot use your normal decoding techniques to actually understand the other person and read how the other person receives your information. And we are facilitating that” (Informant ID)*  **COM-B Model: Motivation**  **TDF Model: Emotions**  **Barriers**  **Anxiety/Stress**  The high number of alarms was perceived as very stressful by nurses, especially false alarms and those to be handled within five minutes  Running back and forth to the Virtual Epidemic Centre (office) when engaged with other patients stressed the nurses  However, one explained, partly taking on the responsibility of biometric measuring potentially makes you feel guilty, e.g., in case a time slot passed, wrong figures were transferred, an exacerbation was overseen, etc  All referred to problems making it work and feeling unsure if they did it correctly  *“Scared, what about my real job tasks, here, I do also have my usual tasks to do”(Informant ID 4/438)*  **Burn-out Caregivers**  However, some raised awareness that unintendedly a role as private nurse or secretary could stress relatives to a degree making them sick  *‘What kind of position do you expect the relative to take in such a future?’ Hopefully not a too important position. Hopefully, they do not feel burdened by responsibility. In the beginning, this will happen, because they do not know the extent to which their resource can be used. Because it does not have to be like that [ . . . ] the relative will get a breakdown, and suddenly have to visit the GP to get sleeping medication or be admitted to the hospital, because of stress symptoms”(Informant ID 4/732)*  **Facilitators**  **Comfort**  *(dialogue without equipment)* the dialogue felt more private  *“I think it will mean a lot to the family [ . . . ] Being at home they can see you all the time. You are not at a hospital, it seems less dramatic”(Informant ID 6/644)*  **TDF Model: Reinforcement**  **Facilitators**  **Incentives**  The HaH model gives relatives the possibility of participating in ward rounds.  *“It is not the same walking 50 m down a corridor with many sick people as walking 50 m down my road where I can enjoy nature and so” (Informant ID 14/2050)*  *“I don’t feel well if I don’t get a good night’s sleep. I know that I couldn’t have slept all night if I needed to monitor and transfer the data, but, but when you lay in a hospital bedroom together with other that would like to see the TV, others that would go for a cigarette or drink coffee, and others having to pee and things like that, then, actually, there is never peace and calm” (Informant ID14/1962)*  *Nice not needing to be hospitalised, because I can walk around in my own home, [ . . . ] go to the toilet, arh, and then lay down on the couch, look TV, and I can make me a cup of coffee in the kitchen, yeah, be together with my family. And then, generally, I think, it becomes most people better to be at home than laying in a hospital bed”(Informant ID 6/602)*  *“Here, at home, I am not disturbed by noises in the night, because someone is admitted or transferred to the intensive unit or . . . And more, there is no one in the corridor shouting ’Is there a nurse here, I am going to the toilet?” (Informant ID 11/2544)*  **Prompts**  Some patients found ways to remember these duties, for example, by setting alarms.  **Feedback**  Knowing that the app included an alarm for immediate contact with a clinician made patients feel comfortable, as did the automatic warning of the nurse in case data were skewed or missing.  *Well, I just need to be sure I’m having the oxygen I need. And in case I doubt, are getting nervous or feel that something is wrong, I can push a button knowing that I come through immediately.(Informant ID 11/2646)*  **TDF Model: Goals**  **Facilitators**  **Health seeking behaviour**  *If you want to be healthy and go home again, then, then every positive indication that, no matter if it is the blood pressure or pulse or oxygen, that makes you happy, so . . . It may give you kind of a boost when you see values yourself instead of just having someone else coming by to tell you, yeah? (Informant ID 9/1155)*  *You are quicker up going [ . . . ] because you return to home, you can walk more around and you are joint owner of your disease, if you can say so(Informant ID 1/431)*  **Meeting patients’ needs**  Positive thoughts of being discharged home earlier than expected were motivated by a mixture of strong wishes for leaving the hospital setting and dreams of being at home in their well-known everyday surroundings. ... being home made them feel free to move around inside, go to the toilet without disturbing, pour a glass of water, eat meals they liked when they liked, phone friends, switch the TV on and off as they pleased, and move out. into the garden, etc. It was explained how wearing daily clothes, sleeping in one’s own bed and being undisturbed by clinicians and roommates.  Escaping from hospital routines to socialisation at home was very motivating for patients  **TDF Model: Intentions**  **Barriers**  **Clash with everyday life**  Clashed with the daily practice of putting the phone on flight mode during night-time  *“Matching of expectations with the patient [ . . . ] consent to, that although it is inconvenient, they have to wake up at 4 o’clock in the morning to do the measuring, because we, as professionals, need the data”  (Informant ID 3/82)*  **Facilitators**  **Building relationship**  *“The patient has to be tested after six hours, according to the algorithm. If you had been admitted in a normal way, they would have made an appointment with the patient about, yes but [ . . . ] then ’I will look after you before you fall asleep, and then we do not need to follow up before tomorrow morning‘. So, it is all about, you have to dare to go beyond the actual wording in the manual”(Informant ID 8/1051)*  **TDF Model: Optimism**  **Barriers**  **Wrongly raised expectations**  *“Human being like us, we are lazy (!) so in the beginning I was just like, do I, oh, do I bother to participate? [ . . . ] Since I decided to participate and learned what it implied, I was thrilled. Being at hospital when you start getting better means that you also begin to bore. [..] Keeping an eye on your own oxygen rate and all that, so it was damned funny. But, being scheduled every 4th hour was somewhat, you know . . . (laugh)”(Informant ID 9/1105)*  **Facilitators**  **Faith**  *“You are quicker up going [ . . . ] because you return to home, you can walk more around and you are joint owner of your disease, if you can say so . . .” (Informant ID1/431)*  **TDF Model: Beliefs about consequences**  **Barriers**  **Avoiding negative consequences**  *“I struggled a little with my blood pressure, because, I measured 3–4 times because showed different values, and therefore I doubted if I had been too unrestful, if I should sit or lay down [ . . . ] So, I lay thinking whether the values I send gave the impression that I was more sick than I really were, and things like that”(Informant ID14/1696)*  **Expected loss of control**  *“The patients left will be more complex and require a lot of care”(Informant ID 13/2112)*  **Facilitators**  **Convenient and reliable care expectations**  *“And then I do also believe [ . . . ] maybe it is easier for the relatives because they can follow the disease trajectory and do not have to call the department all the time when they know we are busy”(Informant ID 5/1312)*  **TDF Model: Beliefs about capabilities**  **Barriers**  **Perceived behavioural control**  Prescheduling of wardrounds intervened in doctors’ usual ‘freedom’  However, patients took on the responsibility to various degrees and experiencing inadequate patient cases scared clinicians  Especially, the nurses felt on uncertain ground being responsible for new and unproven procedures out of their hands  *“I don’t know what time the nurse comes and say ’we need to test now’” (Informant ID 11/2674)*  **Facilitators**  **Perceived behavioural control**  The HaH tasks made patients feel more in touch with the disease and better understand biometric figures. Thereby, they experienced a new appreciated feeling of control and responsibility for own health.  In contrast to usual hospitalisation, the scheduling biometric measuring and ward rounds gave patients a much-appreciated feeling of freedom and ‘time on their own’.  Healthcare professionals recognise a need for patient involvement in the CVW to offer more sustainable healthcare services at home  **Empowerment**  *“If you want to be healthy and go home again, then, then every positive indication that, no matter if it is the blood pressure or pulse or oxygen, that makes you happy, so . . . It may give you kind of a boost when you see values yourself instead of just having someone else coming by to tell you, yeah?”(Informant ID 9/1155)*  **TDF Model: Social/Professional Role and identity**  **Barriers**  **Adapting to new roles**  Being your own nurse during night-time was experienced as the most intrusive consequence of the HaH concept  **Facilitators**  **Adapting to new roles**  A discussion of how to prepare relatives as well as clinicians at hospitals and institutions for their new and still undefined roles was requested  *“We have to change mentality among staff and patients, so it gets an integrated part of the definition of being admitted to hospital, that this actually also can happen at home.”(Informant ID 5/1568)*  *You have joint responsibility. You don’t just lay back and let things happen. You join from the beginning (Informant ID 1/399)*  **Established healthcare professionals**  Several suggested a specialised nursing team dedicated to the virtual patients | **14** |
| **Eines (2023)** | **COM-B: Capability**  **TDF Model: Skills**  **Facilitators**  **Competence/experience**  *“When we have competence and skills with using different assessment tools, I think we contribute to faster treatment of patients with exacerbations, and thus reduce the severity of complications’ (Nurse, b:4)”*  **TDF Model: Knowledge**  **Facilitator**  **Knowledgeable staff**  An equal understanding of the assessment scores was also mentioned as useful carrying out follow-up measures.  The GP elaborated on how weekly in-person interprofessional whiteboard meetings contribute to better coordination and continuity in the follow-up with patients with multimorbidity in CVWs, because they exchange information and knowledge and discuss each patient's health condition  ‘*One individual score does not tell everything, but when we all discuss our assessments, we reach better solutions for the patients’ (Physiotherapist, a:5)*  *‘The observations and information shared in the whiteboard meetings help me to ensure better measures*  *in the medical treatment of the patients' (GP, a:1).*  **COM-B: Opportunity**  **TDF Model: Environmental context and resources**  **Facilitators**  **Resources**  Furthermore, the results show that healthcare professionals consider the assessment tools and whiteboard meetings to be useful tools for facilitating IPC  The interviewees reported that NEWS2 was a useful assessment tool to identify patients with acute exacerbations, heart or lung disorder events, diabetes or infections, especially when following up with patients with multimorbidity post-discharge from the hospital. In particular, the nurses shared their experiences about the use of assessment tools as crucial to identifying acute exacerbations and exchanging information interprofessional physiotherapists and the GP highlighted how the assessment scores lead to an objective and standardised starting point for interprofessional discussions  Some of the healthcare professionals also mentioned how they recommend that patients use welfare technologies, such as GPS, memory planners, medical dispensers or oxygen saturation monitoring, increase the health promotion focus among patients with multimorbidity. Some healthcare professionals also talked about how using standardised assessment tools contribute to earlier detection of acute deterioration  The healthcare professionals described the whiteboard as useful for getting information. The participants also stated that the whiteboard visualise the patients' health conditions. he healthcare professionals talked about how the use of the whiteboard leads to a shared understanding of each patients' goals, which helps them to prioritise interprofessional measures more  *‘I feel the whiteboard helps me get information of each patient's situation in the interprofessional discussions’ (Physiotherapist, a:5).*  **Technical/technology use**  *‘I like the way we all suggest use of welfare technology to prevent unfortunate injuries and health deterioration’ (Nurse, c:3).*  **TDF Model: Social Influences**  **Facilitators**  **Positive relationships**  The nurses especially talked about how frequently home visits lead to patients feeling confidence getting healthcare services at home instead of in a nursing home or hospital  This study shows the importance of mutual interprofessional trust and respect, which leads to a common desire to address the patients' needs. The perspectives of all healthcare professionals were considered equally important in the weekly in-person interprofessional whiteboard meetings  **Support**  The nurses also shared their experiences with the patients' next of kin as an important and necessary supporter enabling some patients to continue living at home.  **Interprofessional work**  interprofessional discussions based on the use of assessment tools contribute to a more informal communication in the IPC and increase the holistic approach to follow-ups with patients with multimorbidity in the CVW. interprofessional discussion of assessment scores and medication reviews may be essential to prevent hospital admissions and mortality for patients with multimorbidity.  *‘I feel the whiteboard helps me get information of each patient's situation in the interprofessional discussions’ (Physiotherapist, a:5) “I like the way interprofessional whiteboard meetings contribute to better use of interprofessional competence in planning measures for the patients’ (Nurse, a:2) “The observations and information shared in the whiteboard meetings help me to ensure better measures in the medical treatment of the patients'” (GP, a:1).*  **COM-B Model: Motivation**  **TDF Model: Goals**  **Facilitators**  **Meeting patients' needs**  The GP also talked about involving the patients to offer healthcare services that meet the patient's needs. They have also experienced instances. in which patients' inner motivations have inspired patients to put forth extra efforts to reach their goals  **Health seeking behaviour**  The healthcare professionals highlighted the importance of focusing on activities promoting health to support elderly patients with multimorbidity so they can continue to stay at home  *‘I think we now have a culture for focusing on promoting health when discussing the patients' issues' (Nurse, b:2)*  **TDF Model: Intentions**  **Barriers**  **Discipline**  *‘If someone misses a whiteboard meeting, the absent member must be contacted afterwards, which leads to extra workload especially for the leader’ (Physiotherapist, a:4)”*  *‘We should never underestimate how motivation can be a driving power for the patients reaching their defined goals’ (Nurse, c:1).*  *We respect and listen to each patient's needs. Different healthcare professionals can suggest measures, but they are useless if*  *the patient is not listened to and involved and motivated for the measures or activities' (Physiotherapists, a:4).*  **Facilitators**  **Building relationships**  Most healthcare professionals also discussed the importance of prioritising participation in the whiteboard meetings because fewer members contribute to insufficient interprofessional discussions  **TDF Model: Beliefs about capabilities**  **Facilitators**  **Empowerment**  In all focus group interviews, the healthcare professionals emphasised the benefit of listening to the patients to identify, define and carry out goals and measures for patients with multimorbidity in the CVW.  Particularly, the nurses emphasised that assessment scores gave them more confidence when they need to contact the GP.  *“The patients’ voice helps us adjust the measures in a better direction’ (Physiotherapist, a:4” “We respect and listen to each patient's needs. Different healthcare professionals can suggest measures, but they are useless if the patient is not listened to and involved and motivated for the measures or activities' (Physiotherapists, a:4)” “I agree, without involving and respecting each patient's needs, our suggestions are useless’ (GP, a:1)”*  **TDF Model: Social/Professional Role and identity**  **Facilitators**  **Stakeholders support**  The healthcare professionals emphasised how important it was that the senior management facilitated and supported them in planning and running weekly whiteboard meetings. In particular, the nurses and nurse assistants elaborated on how the role of the CVW leader is significant in coordinating and sharing information among the healthcare professionals in the CVW  *‘The nurse who leads the CVW always asks us what to report to the other professionals’ (Nurse assistant, c:2).*  *I also feel like a part of the IPC when the CVW leader informs us about the conclusion of the weekly*  *whiteboard meeting’ (Nurse assistant, c:5).* | **8** |
| **Gagnon (2020)** | **COM-B: Capability**  **TDF Model: Skills**  **Facilitators**  **Competence/experience**  The nurse manager’s experience in communication and program management was considered an important asset.  Successful implementation depends on the quality of communication with patients/caregivers, regarding recruitment and follow-up  **TDF Model: Knowledge**  **Facilitators**  **Knowledgeable staff**  The team was already familiar with the CLSCs of the region  **Knowledge gathering**  The establishment of home care protocols for patients transitioning from hospital to home care, however, involved significant initial information gathering as well  **COM-B: Opportunity**  **TDF Model: Environmental context and resources**  **Facilitators**  **Mixed models with flexible components**  This program was conceived in a modular fashion, with flexible components that can be adopted and adapted as needed in other contexts.  **Resources**  As a home care program was already established, the VW procedures were built on pre existing home visit procedures. they had human, material, and financial resources at their disposal.  **Standardized Protocols**  Increasingly standardized protocols were developed for identifying and notifying the patient’s family doctor upon ED visit or hospital admission, identifying eligible patients, ensuring post-discharge follow-up. Protocols were also developed and standardized for communicating with family physicians, home care services, and patients/caregivers.  **TDF Model: Social Influences**  **Facilitators**  **Positive relationships**  The team was already familiar with the CLSCs of the region. The team members had experience working together  **Communication channels**  Gradual development of communication channels with family physicians and CLSCs  **COM-B Model: Motivation**  **TDF Model: Goals**  **Facilitators**  **Evaluation research**  The VW team also took part in evaluating the research examining the program’s processes and impact on readmission rates and length of stay. This involvement in research provided additional impetus to reflect on practices, standardize protocols, and collect indicators Whenever issues or gaps in care were identified, they discussed how these should be overcome and who should be responsible  **TDF Model: Social/Professional Role and Identity**  **Barriers**  **Facilitators**  **Established healthcare professionals**  (Building on established homecare programme) their roles were well-established  **Adapting to new roles**  Continuous negotiation and redefinition of roles  **Stakeholders support**  Positive reception was considered to have greatly facilitated its support from stakeholders (administrators, clinicians and patients/caregivers) and uptake at the JGH. The program was well-received at the outset by health care providers and patients/caregivers alike, and this reception was considered to have greatly facilitated its support from stakeholders (administrators, clinicians and patients/caregivers) and uptake at the JGH | **6** |
| **Harel (2024)** | **COM-B: Capability**  **TDF Model: Knowledge**  **Barriers**  **Eligibility Criteria/Procedural Guidelines**  Another perceived weakness was that this service is not for everyone, due to home and family conditions, geographic proximity, and clinical fit.  AHaH requires careful patient selection—clinically, demographically, socially, and geographically. A patient who does not meet the criteria is excluded from AhaH.  *“The stipulation of 30 km distance from hospital, and limiting AHaH to adults, might cause many potential clients to be lost” (pag.3)*  *“It is difficult to understand whether someone is suitable for AHaH,”(pag.3)*  *“Now, they can see the entire picture of the patient, the continuity of care.”(pag.4)*  *“The general physicians’ workload prevents them from making home visits or they cannot decide whether a patient needs hospitalization, so they refer them to AHaH”(pag.4)*  **Facilitators**  **Knowledgeable staff**  Staff are exposed to what happens in the home, holistic, continuum of care, novelty in internal medicine, variety, and experience for staff  *“More and more general practitioners are aware of the additional benefits and service options”(pag.3)*  **TDF Model: Skills**  **Barriers**  **Assessment accuracy**  *“If the patient needs a CT scan or lab work, we need to take them to the hospital.” “I may miss something because of limited testing”(pag.4)*  **COM-B: Opportunity**  **TDF Model: Environmental context and resources**  **Barriers**  **Resources**  A weakness presented primarily by the HMO representatives was the limited access to on-site testing, imaging, and telemedicine, which may indicate that AHaH provides fewer supportive services compared to hospitals  Given the financial incentives we referred to previously, this could be perceived as a new form of cream skimming and exclusion.  *“Financial considerations threaten the initiative”(pag.4)*  *“Even if you are efficient, you still need to travel from place to place”(pag.4)*  *“When it comes to human resources, the resources for all the health professions are limited”(pag.4)*  **Standardized Protocols**  This was followed by a concern, expressed by both groups, of the risk of a patient not being monitored 24/7 and the need to develop additional standards and quality tools for home care.  Hospital and HMO representatives thought hospitals were neither well integrated nor reimbursed sufficiently within the national model of AHaH.  It might be worthwhile to develop a checklist for emergency departments to gauge the true at-home treatment capacity in this regard  **Facilitators**  **Resources**  Investment is needed to develop a robust in-house AHaH service. Developing more sophisticated services for a varied patient population requires investments in technology and staff  *“We need someone dedicated to home care,”(pag.3)*  *“Caregivers at home should be compensated”(pag.3)*  **Standardized Protocols**  It might be worthwhile to develop a checklist for emergency departments to gauge the true at-home treatment capacity in this regard  *“Risk and quality aspects need development” (Pag.3)*  better communication between hospital and community.  *“the family doctor is involved during the hospitalization and does not just get a report at discharge”(pag.3)*  **COM-B Model: Motivation**  **TDF Model: Emotions**  **Barriers**  **Burn-out Caregivers**  Whereas other respondents were leery of the burden on caregivers.  **Facilitators**  **Comfort**  *“Patients stay in their normal environment,”(Pag.3)*  *“Treatment is provided on their schedule,” (pag.3)*  *“I do not remember a violent incident, I have more time, I am a guest, I am respected”(pag.3)*  **TDF Model: Reinforcement**  **Facilitators**  **Incentives**  Some respondents mentioned AHaH as encouraging patients and family to take more active roles in the healing process  Our fundings reflect the notion that there is potential savings on an organizational level  *“If they had an incentive, they would cooperate more.”(pag.4)*  *“An opportunity to give staff extra work and extra hours”(pag.4)*  **TDF Model: Beliefs about consequences**  **Barriers**  **Expected loss of control**  Worth mentioning is the concern that doctors will refer patients to AHaH when they are not sure what they need  **TDF Model: Social/Professional Identity and role**  **Barriers**  **Stakeholders support**  *The healthcare system is not united behind the concept. “The Ministry of Health does not include hospitals in the equation” (pag.4)*  *“We need to build a new practice philosophy”(pag.4)*  **Adapting to new roles**  AHaH requires medical staff to work with caregivers who are not professional healthcare providers. Laymen assuming professional roles may result in mistakes and potentially risking patient safety  F*amily member becomes part of the treatment staff (pag.3)* | **7** |
| **Herlitz (2023)** | **COM-B: Capability**  **TDF Model: Memory, attention and decision process**  **Barriers**  **Poor health/tiredness**  The main barriers (for both modes) reported by patient and staff interviewees to patients submitting readings were feeling too poorly, tired or forgetting  *“In the beginning I found it [entering the information] difficult because I was poorly [..]” (Site C, patient 6)*  **Cognitive capacities**  Additionally, a few staff interviewees reported that phone calls were labour intensive and required a larger workforce and manual data entry.  However, several patients and staff reported issues related to monitoring applications (‘apps’ hereafter), particularly during set-up because they required downloading and remembering log-in details  **TDF Model: Skills**  **Facilitators**  **Training/learning**  Supporting technology skills development through over the-phone or face-to-face support.  *“[..] I’m not good at things like that. I found it easier to do after a while.” (Site C, patient 6)*  **TDF Model: Knowledge**  **Facilitators**  **Knowledge gathering**  Phone calls were essential in both models for staff to gain comprehensive knowledge of their patients’ condition. Staff across sites gathered information on patients’ medical history and social needs at the referral and triage stages, which they referred back to during monitoring. Phone calls were useful for gaining information about social circumstances and mental health, sometimes prompting referrals to other services.  **Eligibility Criteria/Procedural Guidelines**  However, seeking further help if they had concerns about their health was significantly easier for patients using tech-enabled modes, although, differences might reflect differing characteristics of patients using the modes  **Patient and family knowledge**  *“What we found initially was that [expectation of submitting twice a day] wasn’t explained fully and then we were having to chase them a lot for readings. So that is really explicit at the start now that… almost like as daft as it sounds like a verbal contract. You know, ‘We will do all of this however, what we expect of you is you know readings twice a day to be compliant’.” (Site I, staff3)*  **COM-B: Opportunity**  **TDF Model: Environmental context and resources**  **Barriers**  **Technical/technology use**  However, qualitative findings indicated that some staff were frustrated by the length of time it took tech providers to make changes to platforms and that tech-enabled platforms could be improved to better support clinical and operational needs.  to improve the usability and interoperability of tech-enabled platforms for clinicians through co-design and testing  *“At the peak we were on I think hundred and ninety-six on the service… and each of them were submitting three or four points of data, three times a day…. that’s well over two thousand bits of data coming in a day. There’s just no way manually we would have been able to do that.”(Site 1, staff G)*  **Facilitators**  **Mixed models with flexible components**  Mixed models were considered by staff to be more scalable than analogue-only models. Service leads from three sites reported that they had adopted tech-enabled platforms to ensure the service could cope with a high volume of patients, and staff from six Tech enabled sites highlighted efficiency gains from data being entered by patients in real-time and not having to call every patient.  Across sites, regardless of modality of the model, staff found it useful to adopt a traffic light (red, amber, green) system for prioritising care. Staff reported monitoring patients as statistically significantly easier for mixed models compared to analogue-only models.  **Technical/technology use**  Most patient interviewees across sites reported that it was “easy” or “straightforward” to relay readings whether by tech-enabled or analogue modes. Some patients thought that the simplicity of the tech enabled systems was its strength. Patients using tech-enabled modes were more likely to report that recording readings (p = 0.009) and providing readings (p = 0.001) to the service were easy compared to patients using analogue-only modes. However, differences might reflect differing characteristics of the patient groups  A small number of patient interview and survey respondents suggested improvements to app functionality: easier navigation, being able to access visual charts or tables of all readings entered, improvements in the system parameters (e.g. allowing decimal points for temperature), and the ability to record other symptom  to encourage digital inclusivity for patients by developing user-friendly platforms through codesign and testing, providing home internet and technology  **TDF Model: Social Influences**  **Facilitators**  **Positive relationships**  Staff and patients from mixed model sites mentioned that continuity of staff making the calls was valued as staff could build a rapport with patients and better judge their recovery progress. Staff members (across both models) highlighted that on the phone patients were more likely to mention other medical symptoms that might require attention  **Support**  Many patients and staff highlighted that family members or carers had assisted in relaying readings; particularly patients who lacked confidence in their digital skills, patients that were unwell or had communication difficulties.  Two sites offered face-to-face support at onboarding for this. *(Help with readings)*  opportunity for patients to discuss symptoms with a clinician reassured them and prevented patients from unnecessarily contacting emergency services or encouraged deteriorating patients to go to hospital when they were afraid  *“Many of the patients when we come across… live on their own. So, it’s not just their health it’s the… wellbeing of the patients as well… There are many times where I have done the referrals… and provided more support for them, for food and other medications and things like that”(Side D, staff 4)*  *“[..] then I had a member of my family to help me and then it was just easy to do [..]” (Site C, patient 6)*  **TDF Model: Reinforcement**  **Facilitators**  **Feedback**  Patient interviewees using tech-enabled modes reported that being contacted by staff if they had missed readings gave them a sense of security that someone was watching over them  *“So a lot of [patients], when you speak to them, and you say, ‘Actually look, your levels were 92. I’ve exerted you for a minute they’ve dropped down to 89, can you hear yourself gasping on the phone?’ They say, ‘Yes.’ And I say, ‘Well that’s really serious you do need to call an ambulance.’ And then they will come around to it.”(Site A, staff 4)*  **TDF Models: Beliefs about capabilities**  **Barriers**  **Perceived behavioural control**  Most patient interviewees did not recall being given a choice about the mode in which they could submit readings | **8** |
| **Jessup (2022)** | **COM-B: Capability**  **TDF Model: Memory, attention and decision process**  **Barriers**  **Cognitive capacities**  Rapid implementation requirements meant that initial staffing levels were still not adequate to meet the demands of the service. Staff engaged early reported feeling overwhelmed by the volume of patients  Staff reported that patients (and sometimes even the staf themselves) struggled to understand the diference between the work being carried out by the two agencies, or to recognise who was contacting them, and some reported that they felt burdened by the number of contacts they received.  *‘Thank you for the daily phone calls to see how my husband was… but there were too many phone calls everyday from "everyone’(pag.8)*  **TDF Model: Skills**  **Facilitators**  **Communication**  Having access to interpreters to assist with translating information for patients from migrant backgrounds was essential, and staf often had to provide clarifcation of misinformation in other languages  Staff identified that this skill mix meant there was always someone in the team who could answer a question if another team member was uncertain  It further reinforces that a low technology, high touch approach provided by skilled clinicians operating a call centre is both efective and highly valued by staf and patients alike  **TDF Model: Knowledge**  **Barriers**  **Patient and family knowledge**  *‘There’s a lot of people who don’t know what to do. Information is very limited so even when we tell them to do this and that, sometimes they would get surprised and go “oh I can go out” and I say no*  *because you’re a close contact of this patient so basically you need to be home as well until that patient is cleared. There was some confusion…’ – Interview 7*  **Facilitators**  **Knowledgeable staff**  Rapid implementation of this service was made possible by the ready access to experienced and knowledgeable clinically qualifed staf who had been furloughed from other active clinical roles within the health service  *‘We focused on [people] who don’t speak English and got a person … to interpret. Sometimes one of the family members interprets and that is not appropriate so we provided telephone interpreting services and [translators] locally through Northern Health. That worked very well.’(pag.9)*  *‘The ED and ICU guys understand that acute medical deterioration, but then people like physios and other allied health who work in the community understand the broader contextual needs from a social wellbeing point of view or access.’—Interview*  *2*  **Eligibility Criteria/Procedural Guidelines**  Staff reported that an approach that allowed health care providers within geographical areas to provide all monitoring services would be an improvement  **COM-B: Opportunity**  **TDF Model: Environmental context and resources**  **Barriers**  **Resources**  *‘One of the memories that I have is when we started it was right sort of as that peak was really hotting up and we had three staf at that point.’ (Inadequate staffing)(pag.7)*  **Facilitators**  **Resources**  The DoH provided a specific funding stream to the health service to support establishment of the service  One of the key success factors for rapid implementation was being able to draw on existing staff as a resource  The service would have benefited from more planning time, and onboarding staff in a consistent manner  Implementation in other settings would beneft from [..] shared resources [..]  **Mixed models with flexible components**  The service adapted in response to feedback from patients and staff working in the service, with additional features added. This included text messages as an alternative to telephone calls for those at low risk of developing complications  Consistently reported across all interviews, the greatest challenge experienced by staff was the fragmentation of services and division of roles between the healthcare network (NH) and the centralised services at DoH  One of the reported benefits of running a home monitoring service within the hospital setting was the ability for improved co-ordination of care. Patient management and clinical systems were also linked, so medical staf managing deterioration had access to information about the patients’ health over the preceding days.  *‘[There was] a gap between us and the department [DHS] …we have no [ability to provide] clearance so the patient was still hanging on between us.’ (External policies and incentives)* (pag.7)  **Standardized protocols**  To help facilitate this rapid establishment, key implementation tasks were divided up across four senior personnel within the team, referred to in the interviews as a ‘command centre’. Tis ‘command centre’ division of labour was allocated across the following: workforce, telecommunication, patient management systems and policies and procedures. Telephone systems were set up in a call centre style. with a central phone number that patients could ring to access support  Finally, a number of staf also reported that duplication of care could also occur across health services, with a number of health services potentially being involved with a single household, depending on the test site for individual members  *‘They created a shared drive which the majority of our information went into; introduction packages that we sent to patients [etc.]. The policy itself is on Prompt [hospital intranet], templates we used when speaking with patients, so that its consistent … was emailed … and was on the [shared] drive so you could access it yourself, and as they got updated, they emailed all of us so that way if there were any changes we knew straight away.’ (pag.7)*  *“We had staff starting on different days. This meant I kept being taken away from the call centre to train the new staff when we were really busy. This could be improved by having staff all start on the same day.’ (pag.7)*  *We would call the emergency department if the patient was coming in just to let them know that a Covid + patient was coming in.’ (Improved coordination) (pag.9)*  **TDF Model: Social Influences**  **Facilitators**  **Support**  The main reported strength of the service was its ability to adapt to provide personalised support and education for patients. This allowed staf to build rapport and trust  All staf that were interviewed reported they felt well supported by their peers in the service and by the leadership team  *‘If it was to happen again I think each healthcare service should be responsible for their local area but there would need to be better co-ordination between health services.’ (pag.9)*  *“The service was the best service- because we had no friends or family support, you gave us good advice on how to isolate to prevent the spread of the virus”- Patient survey respondent 168*  **Positive relationships**  *“Initially I was very hesitant to work here because I’ve worked in ED for almost 10 years and I hate change but because ED is not safe for me at the moment, I was offered… I mean they wanted me to get redeployed in this job and initially I thought oh my god, I don’t know I can do it. From day one they have been welcoming and I didn’t get intimidated at all because my suggestions were always welcome, they would always listen and stuff so yeah I’m just…I’m thankful that I have been redeployed here.’(pag.10)*  *‘When I called to talk to him his wife answered the phone and she said he can’t talk at the moment, he’s really sick and I’m trying to get him to the hospital, can I talk to you later on. I said no I can actually help you, do you need some help? She explained the situation … that she was trying to get him to hospital and she couldn't. I offered to speak to her husband and managed to have bit of a conversation with him and built a rapport. I built up enough trust with him that he then let me call an ambulance for him.’(pag.8)*  *I had one patient that I’d been following up every day for a good 4 or 5 days and one of the days that I rang her, probably*  *about 15 min later than normal, she said “I’ve been waiting for your to call. You make my day.”’*  **COM-B Model: Motivation**  **TDF Model: Emotions**  **Barriers**  **Anxiety/Stress**  The rapid change in workforce roles meant some reported feeling nervous initially  **Facilitators**  **Feeling important**  *‘I think it goes to that idea of people having meaningful work, and I am important’ (pag.10*)  **TDF Model: Reinforcement**  **Facilitators**  **Incentives**  *‘We were able to redeploy staf who were pregnant or who had health concerns that would put them at risk if they staying working on the front line.’(pag.7)*  **TDF Model: Intentions**  **Facilitators**  **Discipline**  Implementation in other settings would benefit [..] and commitment  *‘We had huddles twice a day with the group as well as the leadership team… it was through those huddles we [made] continual changes to that procedure.’(pag.7)*  **TDF Model: Beliefs about capabilities**  **Barriers**  **Perceived behavioural control**  Tose responsible for training new staff reported feeling frustrated by having been removed from monitoring patients to onboard new staff at irregular intervals  **Facilitators**  **Empowerment**  (Staff) they were given opportunities to contribute to the development and the direction of the service (including through improving the telephone scripts, and policies and procedures)  *‘I think it goes to that idea of people having meaningful work, and I am important’ – Interview 4*  **Perceived behavioural control**  Our findings suggest that providing staf with a sense of control over decisions around redeployment/ role transition, and seeking staff contribution towards decision  , led to a high sense of satisfaction amongst staff and reduced the risk of transition shock  **TDF Model: Social/Professional Identity and role**  **Facilitators**  **Stakeholders support**  Leadership engagement was considered essential to the success of this service, all the way from the hospital executive to the leadership within the team itself  Implementation in other settings would beneft from strong leadership and [..] and a single point of contact for patients to provide support, education and manage clinical deterioration  *‘I played to people’s strengths or areas and used a command centre approach [to service establishment].’(pag.7)* | **10** |
| **Lee (2022)** | **COM-B: Capability**  **TDF Model: Skills**  **Barriers**  **Assessment accuracy**  Challenges with virtual assessment accuracy and rapport  Lacks observation level of inpatient care  **Facilitators**  **Training/Learning**  Encouraging practice of coping strategies in real-life situations  For referring GPs and GP VMOs working in the clinic, key benefits highlighted were skills and knowledge gained from direct access to specialist expertise, quality of the information exchanged and the efficiency of multi-disciplinary learnings received during the pre-clinic and joint consults  **TDF Model: Knowledge**  **Facilitators**  **Knowledgeable staff**  For referring GPs and GP VMOs working in the clinic, key benefits highlighted were skills and knowledge gained from direct access to specialist expertise, quality of the information exchanged and the efficiency of multi-disciplinary learnings received during the pre-clinic and joint consults  *“It allows providers to see patients in their home environment and make sustainable treatment plans” (pag.6)*  **Eligibility Criteria/Procedural Guidelines**  Risk of shifting individuals who require inpatient care being shifted to virtual due to bed shortages  Standardized protocols for admission, care delivery, and discharge  Providers highlighted clinician presentations where treatment is more suited to a supervised/closed environment, such as addictions, active suicidal intent/other safety concerns, mania and psychosis, and crises that are a result of the individual’s environment (eg, intimate partner violence, problematic relationships).  Providers stated that allowing patients to remain in their homes acted to reduce some of the common patient barriers to typical care [...] physical distance from the care site, disabilities [..]  *“Unfortunately, the use of virtual services and the requirement of technology excludes a significant portion of our client population, including those who have lower [socioeconomic status] or experience homelessness”(pag.6)*  *“[Virtual ward] does not work for patients whose acute crisis presentation had to do with their environment–you can’t always send the patient who [overdose]’d after an argument with tumultuous partner right back to that environment and call them the next day”(pag.6)*  *“A concise procedure/process in writing regarding what to do if there is no contact with a client; how long/how many attempts [to] make”(pag.6)*  **TDF Model: Memory, attention and decision process**  **Barriers**  **Cognitive capacities**  *‘Thank you for the daily phone calls to see how my husband was… but there were too many phone calls everyday from "everyone’(pag.8)*  **COM-B: Opportunity**  **TDF Model: Environmental context and resources**  **Barriers**  **Resources**  Lack of adequate staffing leads to limited capacity and increased wait times  Lack of adequate equipment due to limited budget  *“Sometimes you are stuck trying to manage a very complex case without any of the actual supports you would’ve gotten in the in-person setting”(pag.6)*  **Technical/technology use**  Additionally, the issue of access to virtual-enabled devices (ie, telephone, internet) was identified.  For virtual, they felt sorting out technical issues during the appointment cuts the actual consult time and suggested prior training of GPs for virtual connection might preserve timings for consultation. In some instances, patients in virtual consultations were put on hold to allow for internal discussions, for example GP VMOs having brief discussions with the supervising endocrinologist  *“Technical issues can be frustrating”* (Pag.6)  **Facilitators**  **Resources**  Creating a smoother transition to the community following discharge  **Standardize Protocols**  Providers identified several changes they felt were needed in the administration of the vWard process, and having a standardized protocol for admission, care delivery, and discharge.  Optimization of strategies required for scheduling appointments  Providers also felt that the length of stay should be extended for patients needing longer periods to stabilize their crises  **TDF Model: Social Influences**  **Facilitators**  **Positive relationships**  Creating and building satisfactory relationships with the embodiment of this new service  Facilitating family involvement in care  **Support**  Providers identified the vWard’s immediate and daily check-ins (for support, monitoring, and early detection of deterioration) and ease of medication support as key factors that facilitated stabilization of acute mental health crise  Facilitating family involvement in care  Providers stated that allowing patients to remain in their homes acted to reduce some of the common patient barriers to typical care [..] stigma  **Interprofessional work**  Lack of typical inpatient interprofessional supports (eg, social work, nursing)  **COM-B Model: Motivation**  **TDF Model: Emotions**  **Barriers**  **Anxiety/stress**  *In-person appointment challenges “…I can’t sit in a car for too long...because sometimes I get anxiety…”(pag.6)*  **Burnout Staff**  Additional responsibilities contribute to staff burnout  *“Adding it on to an already very busy service can overwhelm health care providers and contribute to burnout/resentment of the work”(pag.6)*  **TDF Model:Reinforcement**  **Incentives**  *“This provides individuals with the opportunity to continue with their daily activities and/or remain in their personal environment and still attain support”(pag.6)*  **TDF Model: Intentions**  **Barriers**  **Discipline**  The virtual aspect of care may be “too convenient” in some cases and lead to disengagement.  **Facilitators**  **Discipline**  Patient must be self-motivated and engaged with care (ie, can be difficult to connect)  **TDF Model: Beliefs about consequences**  **Barriers**  **Avoiding negative consequences**  *“It’s easy for someone to ‘tune-in’ via Zoom, but also ‘tune out.’ Virtual lacks accountability that one would have with in-person stay”(pag.6)*  **Facilitators**  **Convenient and reliable care expectations**  Providers identified that having vWards available increased the number of options and flexibility of care that they could recommend to patients by offering increased hours in which appointments could occur, different types of care options, and various communication modalities (phone, videoconferencing, virtual resources).  Providers liked having the option to work remotely within the vWard.  **Avoiding negative consequences**  Patients who clinically require inpatient care may opt for virtual (eg, due to social anxiety)  **TDF Model: Social/Professional Role and Identity**  **Facilitators**  **Adapting to new roles**  Providers stated that allowing patients to remain in their homes acted to reduce some of the common patient barriers to typical care, with stated examples including caregiving/work responsibilities | **10** |
| **Kirkcaldy (2018)** | **COM-B Capability:**  **TDF Model: Memory, attention and decision process**  **Barriers**  **Poor health**  The medicines management team participants emphasised how this and the increasing number of patients with memory and mental health issues, impacted upon the time and skills needed to deliver their service  The elderly patients were also slower in their ability to participate physically and mentally  *“We've got a lady who has had to have a little safe to put the blister packs in, you know, that just the carers have got the pin to open it and put in medication and that … [she] has dementia problems, she would overdose … so that was the safety aspect of it.“(FG2P6)*  *“we gave him [the patientan alarm clock to help im remember to take his medication. The patient had schizophrenia. Despite me changing the alarm clock to beep, he’d switched it to voice; and so when the voice went off take his medication, he believed god was speaking to him and he stopped taking all medication!” (FG1P3)*  *“The patients are a lot more complex. They may be not the correct patients to be on the virtual ward from the original conception, but that's the reality that some very complex patients with a lot of issues and problems are the patients that we visit.” (FG1P5)*  *“We're dealing with very elderly people who are a lot slower, even slower to answer the door, slower to walk back to the room, may go and visit the toilet in the middle of the visit [..]”(FG1P5)*  **Cognitive capacities**  Furthermore, the medicines management team identified that the elderly patients were also slower in their ability to participate physically and mentally in a review of their medication, again impacting on the time taken to complete a medication review.  **TDF Model: Skills**  **Facilitators**  **Competence/experience**  Another of the sub-themes that resonated with both teams was medicines optimization. When carried out by the medicines management team this was suggested to not only be of benefit to patients, but also carers  *“it's just a fantastic review of the patient isn't it, that they get and they [medicines management team] highlight issues … obviously as you get older it's all polypharmacy. Sometimes they [medicines management team] can reduce things, or you know, different medication that does the same job with one tablet, or they can put all the tablets together once a day to cut down all the issues with taking them. It's fantastic yeah, it's great.”(FG2P3)*  **Training/Learning**  The multidisciplinary team reported their appreciation of the education given by the medicines management team, acknowledging their role as experts in medicines.\| Education was imparted in both an organized manner through training sessions, and ad hoc through day to day working, side by side on the VW:  *“..and yeah it has been beneficial….some of the meds management team have done like training session and brolught some of the aids, you know, some of the assistive aids you can get, and brought them for us to have a look at. So that’s been really useful” (FG2P3)*  **TDF Model: Knowledge**  **Barriers**  **Eligibility Criteria/Procedural Guidelines**  The distressing nature of some of the situations experienced during domiciliary visits was raised as concern by medicines management team participants.  *“Some of the situations that we encountered with no warning can be quite distressing; a patient’s living conditions..can be quite difficult…to actually sit in someone’s home in that environment or patients’ stories can be quite tragic [..]”(FG1P5)*  **Facilitators**  **Knowledgeable staff**  Both focus groups referred to the advantages of working together within the VW how this affected their specific roels, and the benefits this brought to the patients  Both teams reported frequently finding large quantities of unwanted and unused medication. They explained how they used this as an indicator of non ad-herence, ofyen seeing it linked to adverse events experienced by patients  *“..every time she [the patient] went to the GP, her blood pressure wasn t controlled… so the GP kept increasing her bllod pressure medication, and when we went round we removed four bins bags of blister packs because the lady had literally not taken any medication” (FG1P3)*  *“… they [the patient] had been supplied with it [warfarin] by the hospital, but at that point the GP wasn't aware that the patient was actually taking warfarin, because the GP had actually stopped it [..]”(FG1P4)*  *“I think the multidisciplinary teams are vital because you have all this information, and yet you can go and sit in a multi-disciplinary team meeting, and one of those other disciplines will throw somenthing into the mix and fits the puzzle; so you have a much clearer picture”(FG1P2)*  **Patient and family knowledge**  It was suggested that at times, the provision of aids to assist in the management of a patient's medication was actually more helpful to the patient's carer than to the patient, though it was also thought to protect patients from harm. Both teams believed that patients benefited from being educated about their medication  The medicine management team in particular, saw this *(helping patients understanding their illnesses)* as part of their role, and suggested that this helped to increase patients' adherence to their medication.  *“It's about people's understandings, and we're trying to encourage them [patients] to ask questions and to increase their knowledge around their medication … they say they don't really know why they're taking their medication, and so we will often provide them with an information sheet explaining what the medication is, how often they take it, and what it's used for, and try and stress the importance of taking your regular medication and the reasons behind that, so they're given a rationale for, you know, their compliance really.”(FG1P4)*  **COM-B: Opportunity**  **TDF Model: Environmental context and resources**  **Barriers**  **Technical/technology use**  The medicines management team also described the challenge of accessing a patient's health records held by the patient's GP. The team explained that they had to physically attend the GP surgery to read the notes as they were unable to access them remotely online. The majority of the VW patients were elderly and often had complex medical conditions.  *“We [medicines management team] have had so many IT issues, where, because we're not employed by (NHS Trust) we've not been able to remotely access records (via electronic patient record software system) for the virtual ward patients. So, we have to run round GP surgeries trying to get brief summaries.” (FG1P5)*  **TDF Model: Social Influences**  **Facilitators**  **Interprofessional work**  Joint visits by members of both teams were stated as being especially productive and providing holistic care, as several aspects of a patient's needs could be addressed by one visit.  **Support**  However they voiced the opinions that they were a “close-knit” team and were able to support each other when such instances arose  *“..hard really, to deal with… so we of support each other really”(FG1P5)*  **COM-B Model: Motivation**  **TDF Model: Emotions**  **Facilitators**  **Comfort**  The value of visiting patients at home was raised several times by the medicines management team, who felt that patients were often more confident when being visited in their own homes.  **TDF Model: Optimism**  **Barriers**  **Wrongly raised expectations**  The medicines management team described how occasionally patients' expectations were raised by the VW before they had assessed a patient's needs  **TDF Model: Social/Professional Role and Identity**  **Facilitators**  **Established healthcare professionals**  The medicines management team considered that they frequently acted as a link between the patients' different care providers, supplying and clarifying information regarding the patients' medication | **8** |
| **Ko (2023 a) HAH** | **COM-B: Capability**  **TDF Model: Memory, attention and decision process**  **Barriers**  **Cognitive capacities**  *“Every time… [it was] a different doctor… to some, it might cause some confusion…Perhaps we could have [been] informed that it will be a different doctor.”(Patient, male, 49 yo)*  **TDF Model: Knowledge**  **Barriers**  **Patient and family knowledge**  *“We know how to use [the vital sign monitoring] because we [are] only 50 + [years old], and we understand English. For old people, I think it’s not suitable… they don’t know English. They don’t know how to measure their blood pressure, [and] oximeter…”(Patient, female, 50 yo)*  This model of care will not be suitable for older adults with lower literacy  **Eligibility Criteria/Procedural Guidelines**  Besides domestic helpers, our findings suggest that participants with flexible work schedules were more likely to find CVW manageable.  Close proximity with loved ones This was especially important to patients who “don’t speak any English” or did not know how to use phones to communicate with their family members  **COM-B: Opportunity**  **TDF Model: Environmental context and resources**  **Facilitators**  **Resources**  Participants with flexible working schedules were more likely to find CWV manageable. Conversely patients may find getting support from their workplace challenging when they are admitted to the CVW  **TDF Model: Social Influences**  **Barriers**  **Support**  Patients who stayed alone turned to family members, neighbours, or friends for support CVW was perceived to be challenging for participants without social support or felt that this model [..] and limited social support  Conversely, patients may find getting support from their workplace challenging when they are admitted to the CVW, because they are at home rather than in the hospital  *“[if] I don’t have a helper, I will just admit her [to the hospital] because I can’t cope.”(Caregiver, female, 56 yo)*  **Facilitators**  **Positive relationships**  More assurance can also be provided if participants could receive care from the same doctor  **Support**  The use of the chatbot to enter their vital signs was also deemed manageable. However, some caregivers expressed that they had to help the older patients with the chatbot.  Informal support from caregivers and family members helped to provide food for patients while they self-isolate and ensure surface cleaning and disinfection. Many participants in this study resided in a multi-generational household and thus, “took turns ” with caregiving duties between family members.  In addition, formal support from domestic helpers was important, and was commonly hired in the patients’ households. In some cases, caregivers heavily depend on these domestic helpers to provide daily care for patients, and they would not have opted for CVW without this additional help  Key enablers include social support  *“[My friend] only [stays] a block away…[she will] buy breakfast…If you want to do this…the person has [the patient needs to have] some kind of support system.” Patient, female, 33 yo*)  **COM-B Model: Motivation**  **TDf Model: Social/professional Identiy**  **Barriers**  **Adapting to new roles**  *“We only got the documentation [medical certificate] after the discharge…So that really stresses me… I get pressurized from my employers [asking me] like when am I coming back to work.” (Patient, male, 41 yo)*  **TDF Model: Emotions**  **Barriers**  **Facilitators**  **Comfort**  Given the COVID-19 visitor restrictions in the hospitals, many family members felt that CVW is a good alternative where they could be closer to the patients.  Patients reported being “in the comfort of my home ”, resulting in a better quality of sleep and appetite.  CVW also brought familiarity, which was especially important to a specific group of high-risk patients - the elderly or those with dementia  *“..[the patient] has dementia, [so] she doesn’t recognize a lot of people. Having her at home when she recognizes me, will [has] help[ed] her a lot” [caregiver, male, 41 yo]*  **TDF Model: Reinforcement**  **Facilitators**  **Prompt**  They also perceived that they needed to receive timely access to medical attention in the form of quick response to abnormal vital sign readings, and a 24-hour emergency hotline. To provide more assurance on timely access to medical attention, participants suggested that the chatbot should prompt them as to when the care team would be contacting them, should an abnormal reading be reported  Push notifications sent through the chatbot during a designated time were also helpful  *“…they [the care team] will give me [an] alert that it’s time to send the vital sign information…[I will] key in…my mum’s IC [Identification Card number], then they will offer you [to key in the vital signs in the chatbot], it’s very easy to operate.” (Caregiver, female, 56 yo)*  **Incentives**  Participants felt that it was “very assuring” that essential supplies or services were promptly delivered to them (e.g., nursing services, medication, vital sign equipment)  *“If there is any problem, it can be solved immediately. I don’t even have to hit that [emergency] hotline. As soon as I submit [an abnormal vital sign reading], they [the care team will] automatically call me.”(Caregiver, female, 58 yo)*  **TDF Model: Beliefs about consequences**  **Facilitators**  **Avoiding negative consequences**  Some even found it “safer” compared to the hospital, as they perceived lower infection exposure as compared to the hospital setting  *“...[the hospital was] not suitable for me because I’m under chemo [therapy]... [patients in the hospital] keep coughing...[they were] not wearing masks... So, I chose to go back [home, where] it was safer ...”(Patient, female, 55 yo*)  **Convenient and reliable care expectations**  Easy access to medical attention  **TDF Model: Intentions**  **Barriers**  **Discipline**  While most participants had no difficulties taking vital signs at the designated time, some had to ensure they awoke early (before 8AM) to submit their vitals at the correct time, which they felt required “discipline”. Nonetheless, many suggested that it was manageable and essential to “make sure that the readings are taken” and that the patient’s conditions were “stable” | **9** |
| **Ko (2023 b) VW** | **COM-B: Capability**  **TDF Model: Skills**  **Facilitators**  **Communication**  Clear and detailed communication with such providers was critical to improving the perception of care continuity  **TDF Model: Knowledge**  **Facilitators**  **Knowledgeable staff**  Participants agreed that all care teams needed to be competent to deliver clinical care in a remote and  home setting.  **Barriers**  **Knowledgeable staff**  *“They [3rd-party community service providers] do not know what exactly she [the patient] was treated for, so they would start asking questions, “so what was she admitted for?” …Not very assuring...” [LAR, male, 92 yo]*  **Patient/family knowledge**  *“…it is a new experience since I am not medically trained. So, there will be a situation where I don’t know what to do. So, I don’t want to judge the situation wrongly. A bit of stressful [stress] for me.” [Caregiver, male, 53 yo]*  **COM-B: Opportunity**  **TDF Model: Environmental context and resources**  **Barriers**  **Resources**  Working caregivers preferred to work from home while patients were admitted to HaH. This presented additional challenges of work arrangements or taking time off to be at home. Sometimes, caregivers had to use unpaid time off to care for their loved ones  Nonetheless, the cost was a key factor when enrolling for HaH, and there was a consensus that HaH should be subsidized.  *“…when you are at home, somebody will have to take care [of the patient]. Like in my case, I have to*  *apply for leave, I need to take care of her [the patient] … there is a cost. Cost in the sense that*  *when I apply leave [for work], I got [have] no pay.” [Caregiver, male, 75 yo]*  *“I’m willing to pay more than being hospitalized in the hospital…the convenience and the attention far outweighs*  *that [the additional cost incurred].” [LAR, male, 92 yo]*  *“The main concern will be price, home care, the blood test, MRI [Magnetic Resonance Imaging], are we going to pay? How much is the cost? And how is the person going to pay? Cash or Medisave [governmentadministered medical savings account]?” [Patient, male, 59 yo]*  **Technical/technology use**  In contrast, several other participants highlighted issues with vital signs monitoring, including [..], and instability of transmission of readings.  *“Once I go shower, the patch will not stick again… and you sweat so much, it will come out.” [Patient,male, 59 yo]*  **Facilitators**  **Resources**  The cost was a key factor when enrolling for HaH, and there was a consensus that HaH should be subsidized.  Healthcare financing strategies to ensure HaH out-of-pockets costs affordable which are critical to keeping HaH as an option for patients and families  **Standardized protocols**  While participants had positive experiences with the HaH care team, several others felt that the partnership and communication between the hospital-based care team and 3rd-party medical house-call providers could be improved to increase a sense of care continuity  **TDF Model: Social Influences**  **Barriers**  **Support**  However, the perceived lack of caregiver support and round-the-clock direct supervision from the care team had made some patients prefer staying in a hospital ward.  *“…I think the hospital [was] better. Because there will be nurses all down there [the hospital]. [If I] want to ask something, also can…my wife all make noise already, [asking me] why you cannot stay [in the] hospital.” [Patient, male, 54 yo*]  **Facilitators**  **Support**  Furthermore, it was common for participants to employ live-in domestic helpers that provided additional support for HaH patients. Many households often employed live-in, paid, domestic helpers. During the HaH program, these helpers were frequently entrusted to provide physical caregiving roles, particularly for elderly HaH patients  All participants expressed the importance of having caregiver support during the HaH period. It was common for caregiving duties to be distributed amongst multiple family members living in the same household.  The HaH care team must consider patient’s family members as key stakeholders in the patients’ therapeutic alliance and develop ways to support them better  *“She [the domestic helper] sleeps just outside his [the patient’s] room…he would know how to call if he needs help…She’s [the helper’s] very responsive...” [LAR, male, 92 yo*]  **COM-B: Motivation**  **TDF Model: Emotions**  **Barriers**  **Burn-out caregivers**  Nonetheless, some family members did express caregiver stress and disruption of daily routine during the HaH period  Key challenges reported in this study were the stress and inconvenience caused to caregivers  *“I can’t really sleep soundly, I would also be 3/4 awake, or half asleep…Have to be a bit more wary of her [the patient].” [Caregiver, male, 75 yo]*  **Comfort**  In contrast, several other participants highlighted issues with vital signs monitoring, including the discomfort of the wearable patch [..]Participants also agreed that assurance from the care teams was important, especially in HaH.  **Facilitators**  **Comfort**  Participants also agreed that assurance from the care teams was important, especially in HaH.  *But the fact that he [the patient] is able to be home, to be in familiar environment, that’s more important than the inconvenience that I encountered to answer questions here and during working hours.” [Caregiver, female, 62 yo]*  *“…it’s better…The fact that he [the provider] video called me, I was more assured that oh, maybe it was nothing… it is not something that needed immediate attention.” [Patient, female, 35 yo] “…monitoring the vital signs was important, like knowing that the temperature and knowing that her heart rate and everything was normal. It gives us assurance.” [Caregiver, female, 29 yo]*  *“…comfort, it is intangible right. You can’t put dollars and cents into it…ability to sleep better right, you are*  *close to the things that you are used to…watch TV…go onto the computer. Where else in the hospital, you can’t do all these.” [Patient, female, 55 years old (yo)]*  *“If they [the doctors] need[ed] to update me, they would just speak to me over the video call. So, it is very responsive…very personalized…not intrusive. Sometimes when you want to discuss with the doctor [in the hospital], it’s out in the open.” [LAR, male, 92 yo]*  **TDF Model: Reinforcement**  **Facilitators**  **Prompts**  Participants were appreciative of receiving frequent calls and visits and having providers that were approachable, patient, and prompt in follow-ups  **Feedback**  Most participants felt assured with vital signs monitoring and accepting towards teleconsultation with their providers through video calls, voice calls, and texts.  Participants also agreed that assurance from the care teams was important, especially in HaH. Participants were appreciative of receiving frequent calls and visits and having providers that were approachable, patient, and prompt in follow-ups.  *“Subsequently, every time she [the nurse] came, she would call me and told [tell] me what she has done. So, I can’t ask for more…Because that’s reassuring. I know what was happening at home when she was here.” [Caregiver, male, 49 yo]*  *“…I think initially they [care team] were still uncertain about whether it’s [VSM] functioning and whether the blood pressure data was being sent.… Yeah, I think there wasn’t real-time feedback…so we are not sure whether it is sent over or not.” [LAR, female, 90 yo]*  **TDF Beliefs about capabilities**  **Barriers**  **Perceived behavioural control**  *“When y’all [care team] come ah, sometimes they [my family members] need to pack their mattress and pillow ah. So, morning and weekends Sunday they [are] not working…they want to sleep, 8am then y’all come already… too many people in my house.” [Patient, male, 54 yo]*  **TDF Model: Social/Professional Identity and role**  **Barriers**  **Adapting to new roles**  *“Sometimes the doctors and nurse come, then they come and talk to me… then that means my work schedule need to be adjusted, because sometimes I have meetings.” [Caregiver, female, 35 yo]*  *“So physically, although I am working. Doctor call… nurse call… I’m the one who answer…Quite a lot of the time, I have to work halfway and answer [care providers’] calls.” [Caregiver, female, 62 yo]*  **Facilitators**  **Adapting to new roles**  Patients felt that being in a familiar environment at home allowed them to relinquish the “sick role” associated with hospital care  **Societal pressure**  Additionally, “filial piety”, an Asian virtue of respect for one’s elders, often translates into the family feeling obliged to provide support for members who are sick  **Established healthcare professionals**  The HaH care team must be accessible, approachable, and reassuring, and communicate frequently and timely with patients and their families. Participants also agreed that assurance from the care teams was important, especially in HaH. Participants were appreciative of receiving frequent calls and visits and having providers that were approachable, patient, and prompt in follow-ups.  *“I felt they were professional, friendly, they assured her [the patient]. I really want to commend the group [care team]. I think they were great.” [LAR, female, 82 yo]*  **TDF Model: Intentions**  **Facilitators**  **Discipline**  to actively participate in their recovery  *“When you are here [at home] then…it [health] is your responsibility. Then you are more concern[ed], which is good…you don’t have to rely on someone to do it, you can do it yourself, better.” [Patient, female, 50 yo]* | **9** |
| **Ravi (2024)** | **COM-B: Capability**  **TDF Model: Skills**  **Barriers**  **Communication**  *“I didn’t speak to the doctor face-to-face, because they take it to another level, who takes everything in, all the reports in. And then she comes back and says, ‘this is what the doctor said, and this is how you’ve got to do it” (Pt02). “face-to-face is better… well, then they can tell me what is wrong with me… then they can tell me what they’re talking about in plain English. Not doctor-doctor English”(Pt07)*  **TDF Model: Knowledge**  **Barriers**  **Patient knowledge**  Low awareness *(of educational resources)*  Provider and patient IT skills.  *“Our client base is not really tech savvy [...]”(Clinic staff*)  **Eligibility Criteria/Procedural Guidelines**  Some challenges of vulnerable patients included limited access to transport  It is often challenging to implement integrated care models in clinical practice as the populations they serve often have complex needs, and come from diverse groups, and these kinds of interventions may face many organizational barriers  **Facilitators**  **Educational resources**  *Education Resources* They need to be simple and to meet their limited health literacy levels, including the education bundles  *“The NDSS [National Diabetes Services Scheme] resources are very wordy. I use other ones – Bakers Institute, or ones from Queensland, or I’ve developed my own. Diabetes Australia have good resources. You need things that are visual rather than simple. There are some good exercise resources too”(Clinic staff)*  **Knowledgeable staff**  *“maybe train the GPs before, because my GP didn’t know how to connect…she had to ring someone to see how to do it. I just thought maybe they should know before starting up on Virtual” (Pt0)*  *“Because you get the input and also get the education…that’s a really valuable thing for me as well, so that with my other patients with diabetes I’m getting much more comfortable using the newer types of medication and understanding where they fit” (GP02).*  **COM-B: Opportunity**  **TDF Model: Environmental context and resources**  **Barriers**  **Resources**  Scanning of the CGM sensor requires patients to either use their mobile phone (requiring internet) or via a reader. However, with limited number of readers available, providers recommended that a larger supply of readers would maximise the benefits of the technology for the patient group  Financial barriers to integrated care raised in the broader literature, of accessing appropriate remuneration from Medicare Billing Scheme for participating in joint case conferencing  *“If we could have more of those [continuous] glucose monitors that would be great. They’re a really effective educational tool, raising awareness to trigger self-management and conversations about self-management. Otherwise, this cohort is pretty bad at doing the finger prick thing”(Clinical staff)*  **Technical/technology use**  Huge variations in the type and age of devices used across the GP practices and their network or need to set up for virtual connections. These issues required a lot of technical troubleshooting  Limited access to [..]and mobile data or home internet and meant they were less familiar with the required technology  Participants perceived that various promotional and educational resources designed for patients were not user friendly  *Difficulty using various virtual platforms and devices “I did first video conference for one of the patients, I can connect somehow, but it’s so hard, you know? So three-way conversation. Patient couldn’t hear. I couldn’t hear. Maybe you guys have special software”(GP05)*.  **Facilitators**  **Resources**  Video case conferencing, telehealth concierge and dedicated nurse practitioner are the unique key features that helped with implementation and has proven feasibility of this model  **Technical/technology use**  *I’ve got the information where I need it and also my doctor can, um, access it whenever” (Pt06)*  **TDF Model: Social Influences**  **Facilitators**  **Positive relationships**  Referring GPs felt being part of the team, with a central role  *“[...] You have to call them, and they won’t answer private or blocked numbers. Texting first can really help. Rapport building is key”(Clinical staff)*  **Support**  *Opportunity to engage Self-management support “they would tell me something and then they would ask, you know, what I thought and, did I understand”(Pt06)*  **Interprofessional work**  *“A small multidisciplinary team is an effective way to deal with complex cases. We are all around the table and deliver the care instantly. Not reliant on email or mail – no bureaucratic hold ups. The group is small and can deliver all the care in one day.”(Clinic staff)*  *If the patients goes out to see a specialist and then comes back the care’s much more fragmented, so this, this model provides more of the holistic care… and the patient as seen that the dieticians, or diabetes educator or spoken to them individually, but then they can see everyone working as a team. And I think that’s a positive thing for the patient” (GP02).*  **COM-B: Motivation**  **TDF Model: Beliefs about consequences**  **Facilitators**  **Convenient and reliable care expectations**  Further challenges included referring GPs’ variable engagement with case conferencing and the expectations with scheduling modifications to work around other appointments in their practice  **TDF Model: Beliefs about capabilities**  **Barriers**  **Perceived vulnerability**  Participants who have attended both modalities felt virtual consultations were more in doctors’ language and hard to ask questions  They *(women)* also expressed that it is difficult to handle the responsibility when they are left alone with difficult assessments/decisions  **Empowerment**  Some expressed that it is difficult to be active and demanding while simultaneously experiencing severe uncertainty.  **Facilitators**  **Empowerment**  Participants appreciated involving them in shared care planning, with an opportunity and time to talk about their treatment with the consultants  The women typically viewed HBTM as positive, also emphasizing the potential empowerment of both the pregnant woman and her partner to take more active roles in follow up  According to the obstetricians, discharging women with at-risk pregnancies from the hospital implies a substantial cultural change in how midwives and obstetricians work, more explicitly based on [..] empowering the women to accomplish the monitoring, while simultaneously feeling safe  Additionally, home-monitored pregnant women stressed the importance of being empowered to interpret and act on their measurements, enabling them to cope with the worries and anxiety related to their increased risk  **TDF Model: Social/professional Identity role**  **Facilitators**  **Established healthcare professionals**  Dedicated nurse practitioner are the unique key features that helped with implementation and has proven feasibility of this model  **TDF Model: Intentions**  **Barriers**  **Discipline**  *“With this model we are highly reliant on the referring GP. They usually are not expecting that they need to be present at the time. It’s probably 50–50 between GPs who contribute and those that don’t”(Clinical staff)* | **7** |
| **Rodgers (2012)** | **COM-B: Capability**  **TDF Model: Skills**  **Barriers**  **Competence/experience**  One female participant stated that English was her second language and highlighted concern about reporting her symptoms to the GP, fearing she would convey wrong information and thus receive the wrong prescription  **TDF Model: Knowledge**  **Barriers**  **Patient and family knowledge**  Patients are sceptical, wary or scared of the unknown consequences. This is a natural emotion magnified by the „comfort zone‟ of long-term exposure to pre-admission case management procedures  **Facilitators**  **Knowledgeable staff**  Participants appeared to perceive a difference between the provision of information and how to apply knowledge which had a positive impact on their quality of life. For instance community matron suggested that the patient changes the order of her medication, to improve mobility  Participants also commented on feeling secure by virtue that the community matrons had good medical knowledge  *“You know you can always ask CM and she will always answer you and you have confidence, I know she wouldn’t tell me anything wrong.” “Yes, she will often suggest things that the GP hasn’t thought of you know; her clinical knowledge is pretty wide.”(pag.22)*  **Patient and family knowledge**  The admission process to the virtual ward was mentioned several times throughout the interviews, and reflected how prospective patients felt apprehension about the unknown. What was the ward and why had they been selected? However, this study found that once the process was explained the apprehension disappeared  **COM-B: Opportunity**  **TDF Model: Environmental context and resources**  **Facilitators**  **Resources**  *“She gets everyone to come here, I’m unable to get out, and so we arranged a meeting here with all the professionals in my house”(pag.22)*  **TDF Model: Social Influences**  **Facilitators**  **Positive relationships**  The confidence in the community matron is based on truth and honesty The community matron gave these patients a sense of security,  This patient had a good professional relationship with her matron based on trust and felt reassured this would not happen on the virtual ward  Nine participants gained comfort from the telephone having trust and confidence in the people associated with the service.  **COM-B: Motivation**  **TDF Model: Emotions**  **Facilitators**  **Comfort**  The community matron gave these patients a sense of security, which implies that trust was established  These feelings of comfort were clearly enhanced by meetings with the matron occurring in their own homes which featured strongly in the interviews  One participant captured the essence of reassurance as a result of what the community matron brings to him. His statement referring to “peace of mind” indicates acceptance and clarity in terms of dealing with his long-term condition  *“I feel more confident… she illuminates the situation.” “Well it seems to bring you a peace of mind.” “I am more likely to have come out with any sort of personal worries or thoughts with her than with my family.”(pag.23*)  **Feeling unique**  The patients liked the feeling of being unique  *“it’s more personalized.”(pag.24)*  *“I am regarded as an individual.”(pag.24)*  **TDF Model: Intentions**  **Facilitators**  **Building a relationship**  A willingness to listen and give time helped to build trust and empowered individuals  **TDF Model: Optimism**  **Facilitators**  **Faith**  Trust is the vital ingredient in the recipe of holistic patient care and participants appeared comfortable voluntarily transferring personal information about their condition and circumstances  **TDF Model: Beliefs about consequences**  **Facilitators**  **Convenient and reliable care expectations**  The reliability of the service provided certainty that they were not alone any more as emphasised by one participant  *Well what can I say, if I wasn’t well, I would have to take nearly a mile walk down to doctors and you get there you have to wait and she just knocks at the door and there she is(Community Matron)”(pag,24)*  **TDF Model: Reinforcement**  **Facilitators**  **Incentives**  *“[..]but towards the end of the day it is so worth it – it is 100% worth it to have the oximeters reading every day to know; to understand where you are […] but I still guarantee that 100% it is a very good idea’.(pag.23)*  **Feedback**  *“I know if I ring CM even if she is not available, receptionist (name of clerk) will always tell her and she will always ring me back.”(pag.24)*  **TDF Model: Beliefs about capabilities**  **Facilitators**  **Empowerment**  The community matron had acted as a catalyst to regaining control engendering a feeling of empowerment by inviting the team to his home equipping him with the power to take the lead  Nine participants said they gained confidence, one participant implies that prior to the arrival of the community he was in darkness implying a lack of confidence and powerless to his situation  The findings emphasise the importance of seeking patient and carer input when designing new case management programmes.  **Barriers**  **Perceived vulnerability**  Plus a perceived vulnerability attributable to their illness  **TDF Model: Social/Professional Role and Identity**  **Facilitators**  **Established healthcare professionals**  Seven participants identified that they perceived the community matron as a facilitator and referred to healthcare roles as being on different levels  *“You know you have got the District nurses at one level and then the hospital in the other and she sorts of bridges that gap somehow.”(pag.22)* | **11** |
| **Schultz (2021)** | **COM-: Capability**  **TDF Model: Skills**  **Facilitators**  **Competence/experience**  Additional key themes associated with successful implementation included experienced clinical leadership  **TDF Model: Knowledge**  **Barriers**  **Knowledge gathering**  Barriers to change included the speed of implementation and the large volume of rapidly changing information for a novel disease, with an unknown disease progression  **COM-B: Opportunity**  **TDF Model: Environmental context and resources**  **Barriers**  **Resources**  *“If there was a very large outbreak home monitoring may be required, the small numbers may have given a false sense of security. If there were large numbers simple monitoring would need to be significantly considered.” (Staff #1)*  **Technical/technology use**  *“The introduction of new technology requires a robust process around it, you can’t do it unless you apply a governance process. You run a significant risk of creating adverse outcomes by ‘just popping an oxygen saturation probe in the mail and sending it’. (Staff #7)*  **Facilitators**  **Resources**  *Everything was brainstormed and implemented over a short period of time. It just meant there was not the same level of consultation. If you were planning this at a different time, detailed consultation would have been required, instead it was ad-hoc phone calls and regular Incident Management Team teleconferences. (Staff #1)*  **Standardized protocols**  *The introduction of new technology requires a robust process around it, you can’t do it unless you apply a governance process. You run a significant risk of creating adverse outcomes by ‘just popping an oxygen saturation probe in the mail and sending it’*. (Staff #7)  **Mixed models with flexible components**  A key success factor that supported rapid implementation was the low-tech (telephone-based) model of care, which kept essential service delivery requirements to a minimum  Opportunities for implementation improvements included the timeliness of the information technology platform development, which would have to include enabled electronic medical records, and broader stakeholder consultation. An opportunity for service delivery improvement included dual notification (to the PHU and the virtual ward of a positive result) to reduce the time to initial clinical assessment. A further opportunity for model improvement was the utilisation of visual telecommunication devices and the need to provide selective patient monitoring  Disaster management command and control was identified as the key mechanism for achieving rapid design and implementation  **TDF Model: Social Influences**  **Facilitators**  **Positive relationships**  Staff reported these were overcome by strong teamwork, a culture of trust  **Communication**  Staff reported these were overcome by open communication  **COM-B: Motivation**  **TDF Model: Goals**  **Facilitators**  **Agile Implementation**  Agile implementation was reported by staff to have improved the model  **TDF Model: Societal/Professional role and identity**  **Facilitators**  **Support from stakeholders**  In our experience, the success of virtual wards in a pandemic context relied on strong executive engagement, agile leadership and a willingness from key stakeholders to rapidly adopt and accept new models of service delivery  **TDF Model: Intentions**  **Facilitators**  **Discipline**  *“The early phase of implementation was really just do it, reflect, learn and change because time was so critical, we stood the ward up in 5 hours” (Staff #6)* | **7** |
| **Vindrola-Padros (2021)** | **COM-B: Capability**  **TDF Model: Memory, attention and decision process**  **Barriers**  **Poor health**  Staff reflected that monitoring using an app only model might not be suitable for all populations, as this approach could exclude patients with low levels of health [..]  **TDF Model: Skills**  **Facilitators**  **Training/learning**  Patient and carer training were identified as the key to the success of these models  **Communication**  Good communication between members of the clinical team was identified as a key facilitator  **TDF Model: Knowledge**  **Barriers**  **Patient and family knowledge**  Staff reflected that monitoring using an app only model might not be suitable for all populations, as this approach could exclude patients with low levels and technology literacy  **Knowledge gathering**  There was a lack of published data to support the design of the remote monitoring models and study sites found it challenging and time consuming to collect the desired data, even when using commercially available apps  **Eligibility Criteria/Procedural Guidelines**  Early on, referral criteria and processes were unclear, which led to patients being referred to these models who might have been ineligible in other circumstances. In part, this was caused by evolving criteria for patient referrals  Some patient groups were more difficult to monitor remotely (e.g. homeless community)  **Facilitators**  **Educational resources**  Paper and video patient information (as well as using digital platforms) was very useful to explain the concept of the remote home monitoring models and how to take measurements using pulse oximeter.  The availability of culturally appropriate patient information in different community languages was identified as a key component of patient engagement  **COM-B: Opportunity**  **TDF Model: Environmental context and resources**  **Barriers**  **Resources**  Staff also found it challenging to deliver a seven day service due to workforce availability.  It is important to note that during the first wave of the pandemic, staff were available to play a role in the delivery of care in these models due to the cancellation of elective care and other activities in the NHS. Participants expressed concern that these staff members would not be available during future surges in patient cases  **Technical/technology use**  Technological barriers have been reported in studies of remote home monitoring for other conditions  Staff found it difficult to carry out non-verbal assessments using telephone and video consultation alone  **Standardize Protocols**  The integration of service data with existing patient administration systems was generally poor, and it was not feasible to arrange data sharing between and within sectors in the time available. Additionally, there was no link between NHS Test and Trace systems and the study sites’ referral processes  **Facilitators**  **Resources**  Acute hospitals that had previous pathways in place (i.e. ambulatory care) or digital protocols that could be repurposed by IT teams were able to set-up these models at a quicker pace.  The sustainability of these models during subsequent surges in patient cases and for other conditions will require more stable flows of funding  **COM-B: Motivation**  **TDF Model: Emotions**  **Facilitators**  **Comfort**  Personalised support might be required to avoid patient anxiety and reach those who may be difficult to monitor remotely  **TDF Model: Societal/Professional role and identity**  **Facilitators**  **Support from stakeholders**  Implementation was facilitated by the active role played by dedicated clinical leaders in establishing the remote home monitoring models. Significant support and ‘buy in’ from senior management within acute trusts and across Clinical Comissioning Groups (CCGs) to set up the models was documented across all sites  Lack of administrative/project management support and resources meant that essential equipment such as pulse oximeters could not be obtained quickly | **6** |
| **Walton (2022)** | **COM-B: Capability**  **TDF Model: Behavioral regulation**  **Facilitators**  **Action planning**  Interview findings supported this by highlighting the importance of developing a routine  **TDF Model: Memory, attention and decision process**  **Barriers**  **Poor health**  Interview findings indicated that patients in poorer health (e.g., due to COVID‐19, other health conditions) found it harder to engage with the service. For example, many participants spoke about feeling too unwell due to COVID‐19 (often during the first few days of the service) and therefore they were unable to engage with monitoring behaviours such as taking and recording readings. Some patients and carers spoke about having other health conditions that made it difficult to engage with monitoring behaviours. However, within the interviews, many barriers relating to physical health were identified, including feeling too poorly/not in the right frame of mind, sleeping a lot, having health conditions that made it difficult to monitor, difficulties hearing, difficulties getting to the telephone and difficulties with eyesight  **Cognitive capacities**  The amount of information received was sometimes reported as a barrier in the interviews (e.g., too much, too little or contradictory and confusing information).  **Facilitators**  **Cognitive capacities**  *“Well I mean. It's quite straightforward isn't it. You just put it on your finger and let it settle down and read the figures off. Pulse‐pulse and oxygen levels. So no, I didn't find it complex at all’. (Site C, interviewee 2)*  **TDF Model: Skills**  **Barriers**  **Competence/experience**  Additionally, barriers to seeking further support/escalating care were identified, including  ([..] difficulty communicating)  **TDF Model: Knowledge**  **Barriers**  **Knowledgeable staff**  Some felt that the service was narrowly focused on managing known symptoms of COVID‐19, which did not always suit those with other symptoms, health conditions or who required wider support  *“I think somebody should maybe discuss some of the other things. To me, I got the impression that as long as I as breathing and my oxygen levels were reasonable, that is all they were interested in. Where there were other things that I was a bit concerned about which I don't think were discussed unless I brought it up’. (Site J, interviewee 1)*  **Patient and family knowledge**  Uncertainty around interpretation of readings and thresholds meant that some patients and carers were hesitant to self‐escalate their care. However, interview findings indicated that knowledge was a barrier for some participants (e.g., relating to understanding/interpreting information and equipment, language barriers, not knowing how to fill out diary/complete readings/escalate care or when to call for help)  A lack of knowledge of how to complete the activities (e.g., a lack of knowledge of how to escalate care or what the thresholds for escalating care are) limited engagement  **Facilitators**  **Patient knowledge**  A majority of survey participants felt that knowing what to do helped them to engage with the service.  Survey and interview participants reported knowing why the service was important and these positive views helped them to engage with the service  **Knowledgeable staff**  *I think somebody should maybe discuss some of the other things. To me, I got the impression that as long as I as*  *breathing and my oxygen levels were reasonable, that is all they were interested in. Where there were other things*  *that I was a bit concerned about which I don't think were discussed unless I brought it up’. (Site J, interviewee 1)*  **COM-B: Opportunity**  **TDF Model: Environmental context and resources**  **Barriers**  **Technical/technology use**  However, other participants experienced difficulties with the app, oximeter and technology systems  Some participants felt that there were problems relating to delays in enrolment, limited hours of service and not being able to continue monitoring following discharge  **Standardize Protocols**  Some participants reported that the service was not holistic (did not cover all symptoms of COVID).  **Facilitators**  **Resources**  Continuity of staff was thought to be important  A fifth of survey participants felt that having time helped them to engage with the service  However, a few participants did not have enough time (e.g., those working from home)  Some participants already had their own equipment, which facilitated engagement, and a few participants reported not having the right equipment (e.g., faulty oximeters/not having thermometers)  **Technical/technology use**  [..]the technology was easy to use.  **TDF Model: Social Influences**  **Barriers**  **Support**  A few patients reported feeling that the service was isolating and unsupportive (e.g., they only received a call about the oximeter dropoff/return, but not for monitoring)  Additionally, barriers to seeking further support/escalating care were identified, including ([..] lack of support[..])  However, a small number did highlight that they did not have enough support from healthcare professionals or that they could not get through to a member of the team.  However, a small number of participants reported that lack of support from family/friends was a barrier to engagement  **Communication**  Wanting to be seen face to face  **Facilitators**  **Support**  Survey and interview participants spoke about how support from healthcare professionals helped them to engage with the service. Interview findings highlighted that support helped to understand information, helped with monitoring, obtaining equipment, recording, communication and escalating care. Support was reassuring.  Some patients and carers wanted more support  A quarter of survey respondents felt that support from family and friends helped them to engage. Interview findings echoed this and highlighted that support from family and friends helped with understanding information, collecting the oximeter, doing the monitoring, recording and communicating readings and escalating care  *‘Really they just, in the main I was quite poorly, in fact I would say I was really poorly, it's the only time I've thought I was going to die in my life […] so really in the main my husband dealt with them, I couldn't really be remotely bothered with them if I'm honest and I can't remember what they told me, I don't think they told me a lot’. (SiteI,interviewee 3)*  *‘So my dad was initially involved in I think it was nine days, so the first nine days he took full care of mum to be honest clinically I was involved in a lot of the calls because I think my dad's getting quite stressed. […] so yes, he did the physical side of it. He would do the observations. And then he'd call me first thing in the morning, or he'd drop a text to say these are the observations. I'd call and have a quick chat, knowing the nurse was going to call us. So I guess it was a bit of a joint effort between us’ (Site F, interviewee 6)*  **Positive relationships**  *‘The nurse was very good, can't praise her really high enough. She was a friendly voice to speak to. Fair enough, you know, I've got a bit of a support system, but for somebody who hasn't got that much of a support system around them, I think that friendly voice would go a long way just to, you know, easing their minds’.(Site D,interviewee 5)*  **COM-B: Motivation**  **TDF Model: Emotions**  **Barriers**  **Anxiety/Stress**  [..] monitoring made them feel worried  [..] Found recording burdensome  However, some participants reported a lack of interest in monitoring/ recording, or views that monitoring did not help or made them anxious if the reading was low.  However, some patients and carers spoke about preferring to be in the hospital rather than at home, to feel more secure, feeling scared  *“But there are sometimes, I must admit, sometimes it makes you feel a little bit anxious [.] but then you can leave it – because you want to get things all right [..]2 (Site A, interviewee 1)*  *“I found, to start with I found the text messages useful but the longer they went on the more irritating. I was, I felt like I was chained to the phone and you know and to my equipment. So three times a day is, I know that's necessary to start with but I just felt that maybe twice a day after that might have been better” (Site B, interviewee 3)*  **TDF Model: Reinforcement**  **Barriers**  **Prompts**  Participants identified barriers relating to inconsistency of call timing, amount of calls, not being able to see progress and frequency of monitoring and recording  **Facilitators**  **Incentives**  Some patients and carers also spoke about how the service helped them to monitor their own improvement and that it potentially improved their outcomes.  **Prompts**  However, phone calls helped participants to do readings, and some participants wrote readings on post‐it notes to facilitate memory.  Some participants felt that reminder texts or alerts from an app helped them to engage  **Feedback**  *“And then near the end when I was getting a bit complacent, I was sort of almost well, a couple of times I didn't put them in and they would phone and say, “Are you okay? You've not submitted you reading.” So that was just really supportive, and I said it certainly reassured me’ (Site M, interviewee 1)*  **TDF Model: Intentions**  **Barriers**  **Discipline**  Forgetting to do the readings was a barrier to engaging with the service  *“Really I should have probably rung when the readings were that bad but I didn't. […] And when I did send them through they said, ‘No, get to the doctors now'. (Site C interviewee 6)”*  **TDF Model: Beliefs about consequences**  **Barriers**  **Expected Loss of control**  Patients reported not wanting to go to hospital or seek further support even when advised to by staff members  **Avoiding negative consequences**  Additionally, barriers to seeking further support/escalating care were identified, including worries about going to hospital (due to COVID‐ 19 [..]  *‘Just really, they encouraged me to ring an Ambulance if I needed it. And I wasn't ringing them, because I felt like I was wasting their time, or whatever. I didn't want to, because I was worried they might want to take me in’ (Site*  *B, interviewee 4)*  **Facilitators**  **Convenient and reliable care expectations**  Perceptions that home monitoring was a suitable care package for those with more minor symptoms of COVID‐19.  Reasons for preferring home over hospital [..] was being able to work  **Avoiding negative consequences**  Findings may have been affected by the pandemic context in that data were collected during the height of Wave 2 of the pandemic; therefore, patients may have been more likely to accept remotely delivered services to help minimize risk to themselves, family members and staff.. | **10** |
