# Supplementary material for: Barriers and facilitators to the use of virtual wards: a systematic review of the qualitative evidence
Source: Int J Qual Health Care. 2025 Jul 18;37(3):mzaf065. doi: 10.1093/intqhc/mzaf065 (PMC12342918; doi:10.1093/intqhc/mzaf065)
Supplement: mzaf065_Supplementary_Data [file mzaf065_supplementary_data.zip › INTQHC-2025-02-0056.R2_Characteristics of included studies (Supplementary material 3).docx]

**Supplementary Material 3: Data Extraction Characteristics of Individual Studies**

| **First author (year of publication)** | **Country** | **Study design** | **Data collection method** | **Recruitment context** | **Participants characteristics** | **Sample size** | **Condition/s** | **VW description** | **Quality appraisal** |
| --- | --- | --- | --- | --- | --- | --- | --- | --- | --- |
| Aasbø (2024) | Norway | Qualitative study | Focus group interviews (FGIs) and individual interviews | Department of Obstetrics, Oslo University Hospital and Norwegian Stillbirth  Society | (1) Midwives currently following up hospitalized pregnant women with ongoing high-risk pregnancies (FGI, n= 6); (2) Senior obstetricians whose responsibility includes follow-up of patients with high-risk pregnancies (FGI, n= 3); (3) Hospitalized patients with ongoing high-risk pregnancies (individual interviews, n= 6); and (4) women who had experienced stillbirth in a previous pregnancy but had later given birth to a living baby (FGI, n= 6). | 21 participants (9 healthcare providers and 12 patients) | High risk pregnancies | The women are equipped with a tablet connected with a device for highly reliable, remote CTG monitoring, as well as equipment to measure BP, temperature, and C-reactive protein (the latter relevant for the pPROM pregnancies). The women downloaded a smartphone application to report parameters for in-clinic assessments. The obstetricians responsible for HOME provided all women with a personalized plan for monitoring, including an app with diagnosis-specific questions related to their symptoms, which may help midwives and obstetricians to detect early warning signs. A time schedule is to be provided for how women will submit their readings and how midwives and obstetricians will respond to these | 25 points   - Inadequate theoretical underpinning to the research - No justification for analytical method selected - No evidence that research stakeholders were involved in the research design - Strengths and limitations were poorly descripted |
| Cerdan de Las Heras (2023) | Denmark | Qualitative study | Semi-structured interviews | Department of Pulmonary and Infectious Diseases, Copenhagen University Hospital—North Zealand, Hillerød | 1) Six patients (three male and three female, aged 36–61 years old(mean 46)), five treated for COVID-19 infection, and one for acute asthma)  2) A 25-year-old daughter accompanying her father, 3) two female nurses being the main responsible for the clinical treatment (34/65 years old)  4) Six clinicians from the investigator group (one male, five females, aged 30–61 years old (mean 41)  4) Four doctors and two nurses, three with a Ph.D.) | 15 participants (7 patients, 1 family member and 12 healthcare providers) | Covid-19 | Patients are expected to perform a range of self-measurements (blood pressure, temperature, pulse, etc.) using mobile medical equipment for domestic use. The data are manually sent to the hospital via an app. Additionally, patients regularly perform self-assessment using a short questionnaire. All data are assessed by clinicians at the hospital via a web-based telemedicine platform accessible at any computer at their regular workstations. In the case of signs of clinical deterioration or if data are not registered timely by the patient, an alarm warns the clinician responsible for further actions. Communication between patients and clinicians is supported by either video consultation or regular telephone calls. | 26 points   - No rationale for choice of data collection - No justification for analytic method selected - No evidence that research stakeholders were considered in the research design or conduct |
| Eines (2023) | Norway | Qualitative study | Focus group interviews | CVW (community virtual wards) in Norway | 1) Focus group a: one GP, two nurses and two physiotherapists (age 37–68)  2) Focus group b: two nurses, three nurse assistants (age 36–49)  3)Focus group c: two nurses, three nurse assistants (age 23–54)  4)Focus group d: two nurses (age 31–43).  They all have experience working in CWV | 17 healthcare professionals | Multimorbidity (at least two chronic conditions occurring together) | A Registered Nurse conducts the first home visit within 3days after the patient is registered in the CVW. The nurse invites both the patient and their next of kin to collect data on the patient's state of health, needs and wishes. The first home visit conversation is based on the standardized dialogue ‘What matters to you’, focusing on patient involvement and resources. Additionally, the nurses collect data on the patient's use of medication, results from a 4-m walking test, and nutrition and fall screening. Patients admitted to the CVW are daily followed up by nurses and nurse assistants. Other allied healthcare professionals, such as GPs, physiotherapists or social workers may also be involved. Additionally, different healthcare professionals collaborate in weekly whiteboard meetings to discuss each patient's health status. Halfway through and at the end of the follow-up in the CVW, healthcare professionals conduct systematic evaluations together with the patient and their next of kin. The evaluation is based on the patient's defined goals and the results of retesting of the same tests conducted during the first home visit | 20 points   - Inadequate theoretical or conceptual underpinning of the research - Inadequate description of research setting and target population - Inadequate sampling - Format and content of data collection tool is inadequate to address the stated research aims |
| Gagnon (2020) | Canada | Qualitative study | Group interviews | Urban Montreal hospital (Site 2), and Indigenous reserve (Site 3) | 1) JGH VW team members: VW family physician, VW nurse manager, VW nurse case manager, VW medical resident, a pharmacist, a social worker, and an administrative coordinator.  2) Site 2 participants: the Family Medicine Unit medical director, the Family Medicine Unit assistant director, the Family Medicine Unit department head, and the head of service nurse clinician assistant  3) Site 3 participants: the director of nursing, the home care nurse manager, and a family physician | 14 participants (9 healthcare providers and 5 administrative staff members) | Conditions included heart failure, chronic obstructive pulmonary disease (COPD), gastro-intestinal bleeding, dementia, and diabetes complications (type 1 or type 2). | Interprofessional team, regular weekly multidisciplinary team meetings, and a single point of contact for patients (nurse manager). The entire team participated in weekly multidisciplinary rounds and home care planning. The roles of each VW team member were clearly defined. The nurse manager played a key role in establishing the program, refining protocols, and leading the quality improvement process, provided care coordination among the VW members, patients’ family physician, home care services from community health centers and patient/caregiver. She was the primary point of contact for the patients and their family after discharge and monitored the patients’ progress closely, mainly through telephone evaluation; conducted physical evaluations and adjusted treatment plans in collaboration with the VW medical resident and VW family physician. The pharmacist oversaw medication adjustments. The social worker played an important role in evaluating patients’ social and family environment. The VW family physician and/or resident admitted patients to the VW, reviewed the case, performed home visits to assess the patient’s recovery, regularly assessed patients’ needs, provided guidance to the VW nurse case manager, and adjusted medications when needed. An administrative coordinator provided administrative support and scheduled appointments. | 22 points   - Data collection tool not provided - Inadequate description of data collection procedure - Inadequate description of recruitment - No justification for the analytical method selected |
| Harel (2024) | Israel | Qualitative study | Open-ended questionnaires | Health management organizations (HMO) and general hospitals | 1) Four medical doctors (including two hospital CEOs, one of a virtual hospital that is part of a large hospital), one doctor who oversees an internal medicine ward and an AHaH program, and one doctor who oversees AHaH and a COVID-19 ward).  2) Two registered nurses, one in charge of the “virtual hospital” operations and one in charge of AHAH in a general hospital  3) A chief financial offer was interviewed as well | 14 participants (13 healthcare professionals and 1 administrative staff) | Not mentioned | A service that provides in-home hospital care to patients with complex clinical conditions who would otherwise be hospitalized in conventional facilities due to an acute episode and require 24/7 monitoring and follow-up that is only available in the hospital. | 21 points   - Inadequate theoretical or conceptual underpinning - Inadequate description of research setting and target population - Inadequate sampling - No justification for the analytic method selected - No evidence that research stakeholders were considered in the research design or conduct - Inadequate discussion of strengths and limitations |
| Herlitz (2023) | England | Mixed-method study | Interviews and questionnaires | COVID-19 remote home monitoring sites in England | 1)Six patients  2)Four staff members to interview (including a clinical/operational lead e.g. a senior nurse; a front-line delivery staff; and staff involved in data collection and management, e.g. non-clinical managers) | 10 participants (6 patients, 2 healthcare providers and 2 administrative staff members) | Covid-19 | Patients were referred to the model via community methods or as early discharge from the hospital. They were given pulse oximeters and important information (contact numbers and safety instructions). They were asked to measure blood oxygen saturation with pulse oximeter and other readings at home. Twenty-one sites adopted mixed models whereby patients could submit their symptoms using either tech-enabled (app, weblink, or automated phone calls) or analogue (phone calls with a health professional) options; seven sites offered analogue-only data submission (phone calls or face-to-face visits with a health professional). Readings are reviewed and care is escalated as necessary. Patients were discharged and given safety netting advice and asked to return oximeters within 14 days. | 28 points   - Inadequate choice of data collection tool - Inadequate description of data collection procedure - Inadequate discussion of strengths and limitations |
| Jessup (2022) | Australia | Qualitative study | Interviews | VHC COVID-19 home monitoring service | 1) Five staff were allied health professionals (Physiotherapy, Exercise Physiology and Occupational Therapy discipline)  2) One was medical (Respiratory Physician) 3) Nine were nurses (emergency, pediatrics, cardiac and generalist community and ward trained). Four staff were responsible for development, implementation, and management of the service, four were involved in both the development and implementation as well as provision of service, while the remaining seven staff were part of the direct care team involved only in provision of service | 15 healthcare providers | Covid-19 | Patients were monitored by phone during the acute phase of their illness which was typically up to 14 days. All patients with COVID-19 in the Northeast of Melbourne were managed on this service and were referred following a positive PCR result by the Victorian Department of Health (DoH). A clinical assessment using a script, including a checklist of risk factors that fed into a risk stratification, was used for all patients on entry into the program. Social and welfare needs assessment were also undertaken. Patients stratified as at risk of deterioration were offered ongoing monitoring via a daily phone call; those at lower risk were offered second daily phone calls or SMS. In follow up phone calls, patient symptoms were categorized as stable or deteriorating. For patients with deteriorating symptoms, escalation of care involved either consultation with a medical officer, referral to the general practitioner or hospital emergency department, or calling an ambulance. | 27 points   - Inadequate theoretical underpinning - Inadequate description of data collection procedure - Inadequate justification of selected analytical method - Inadequate discussion of strengths and limitations |
| Kirkcaldy (2018) | England | Qualitative study | Focus groups | Clinical Commission Groups (CCGs) in Northwest | 1) 5 medicines management team members (pharmacists and technicians)  2) 9 from wider multi-disciplinary team (a health trainer, physiotherapist, social worker, occupational therapist, community matrons and district nurses) | 14 healthcare providers | Not mentioned | Dedicated medicines management team, comprising four pharmacists and four pharmacy technicians (1.6 whole time equivalents). The team delivers medicines management support to selected VW patients. definition of VWs by Lewis “… provides multi-disciplinary case managment services to people who have been identified, using a predictive model, as high risks for future emergency hospitalisation. Virtual wards use the systems, staffing, and daily routine of a hospital ward to deliver preventive care to patients in their own homes.” | 23 points   - Inadequate description of research setting and target population - Inadequate sampling to address the research aim/s - Inadequate rationale for choice of data collection tool/s - Inadequate description of data collection procedure - Inadequate strengths and limitations critically discussed |
| Ko (2023) a HAH | Singapore | Qualitative study | Interviews | NUHS@Home programme | 1)13 patients  2)9 LARs. The mean age was 49.8 (SD 12.39). Two-thirds of LARs were female Participants were either Chinese (n = 27), Malaysian (n = 8), or Indian (n = 1)  3)14 caregivers | 36 participants (13 patients, 9 healthcare providers and 14 caregivers) | Covid-19 | Patients were monitored through a biosensor adhesive vital patch for continuous heart rate and respiratory rate readings and Bluetooth-connected blood pressure and oxygen devices. Patients were able to view these readings via a home-placed tablet, synchronized to a clinical dashboard accessible by the care team. Patients who deteriorated were escalated to acute hospitals. | 23 points   - Inadequate theoretical underpinning - No justification for selected analytical method - No evidence that research stakeholders were considered in the research design or conduct - Inadequate discussion of strengths and limitations |
| Ko (2023) b VW | Singapore | Qualitative study | Interviews | NUHS@Home programme | 1)18 patients  2)15 caregivers  Patients discharged from the COVID-19Virtual Ward were invited for an interview. Caregivers of these patients were also invited if they assisted the patient with either the activity of daily living or with medical decision making. None of the participating patients and caregivers were related to each other. The mean age of the patients and caregivers was 54 and 49 respectively | 33 participants (18 patients and 15 caregivers) | Covid-19 | Patients were instructed on how to measure their vital signs using a thermometer, pulse oximeter, and blood pressure machine. Devices were delivered to patients if they did not already have them. Patients or an identified caregiver were enrolled on a chatbot via their mobiles (teleconsultation); the chatbot provided an automated push notification three times a day with an online form to submit their vital signs. Submitted readings were synchronized to an online clinical dashboard. There was a protocol for the care team to follow up on abnormal or unsubmitted vital signs via phone call. All patients had access to the care team 24/7 via a hotline.The CVW care team also collaborated with private on-demand medical house-call services to provide home visits for nursing procedures, such as blood taking, infusion of intravenous fluids or administration of COVID-19 therapeutics, or peripherally inserted central catheter (PICC) flushing | 23 points   - Inadequate theoretical underpinning - No rationale for choice of data collection tool - No justification for selected analytical method - No evidence that the research stakeholders were considered in the research design - Inadequate discussion of strengths and limitations |
| Lee (2022) | Canada | Mix method | Surveys and Workshop | Crisis Response Centre (CRC) and Crisis Stabilization Unit (CSU), two virtual mental health crisis units in Winnipeg | Participants in the provider workshop were: Decisionmaker/manager/leadership, psychiatrist/physician assistant, crisis unit clinicians, CRC clinical staff, and other/unspecified. Patient discharge forms also included patients’ opinions. | 60 healthcare providers and 467 patients | Psychiatric conditions | The lower acuity vWard was managed by a dedicated crisis clinician 7 days a week, usually having training in nursing or social work. The crisis clinician provided 1:1 daily virtual crisis assessment and support, along with optional group classes teaching skills derived from cognitive behavioral therapy and dialectical behavioral therapy. Psychiatric support was available to the lower-acuity beds as required. All patients had access to the 24/7 crisis phone line at the CRC for after-hours support. Medication management, when applicable, was coordinated with the individual’s community pharmacy. vWard admissions were documented in the same electronic patient record used by the CRC, facilitating common access to clinical care details. The target length of stay for the virtual beds was 3-5 days. On average, there were 2 higher -acuity beds and 6 lower-acuity beds available at a given time. | 23 points   - Inadequate discussion of theoretical underpinning - Inadequate sampling - No rationale for choice of data collection tool - Inadequate recruitment data - No evidence that stakeholders were considered in the research design |
| Ravi (2024) | Australia | Mix method | Semi-structured interviews and focus groups | Mt Druitt Community Diabetes Clinic (MDCD) | 1) Adult patients with type 2 diabetes who had attended an appointment at MDCDC via myVC and/or in-person.  2) General Practitioners who had referred their patients with diabetes to the MDCDC and attended a joint case conference.  3)Staff and health professionals working in the MDCDC and co-located in the community health centre. | 28 participants (10 patients and 18 healthcare providers) | Diabetes | Patients are referred to the clinic by their GP. Each case is reviewed by the multidisciplinary team including the four GP VMOs and the supervising endocrinologist from WSD (Western Sydney Diabetes), the nurse practitioner, diabetes educator and dietitian. A full-time nurse practitioner manages the flow of patients booking, pre-clinic consultations including arranging Continuous Glucose Monitoring (CGM), stabilizes insulin and management between appointments and provides diabetes education as needed. The GP VMOs conduct diabetes case conferencing with patients, referring or regular GPs and community health providers. An endocrinologist supervises and co-consults with the GP VMOs, so they are upskilled with in-depth specialist approach and apply the skills in their general practice. myVirtualCare platform, developed by NSW eHealth and Agency for Clinical Innovation, is a custom-built web-based videoconferencing platform that provides secure virtual consultation room and mimics the physical workflow of a clinical consultation | 24 points   - No rationale for choice of data collection - No justification for chosen analytical method selected - No evidence that research stakeholders were considered in the research design - Inadequate discussion of strengths and limitations |
| Rodgers (2012) | UK | Qualitative study | Semi-structured interviews | Not provided | All patients who had been admitted onto the virtual ward for more than three months and who had suffered from two or more long-term conditions. Seven men and four women with an age range of 53-89 years, mixed ethnic background. | 11 patients | Long term conditions | Not provided | 24 points   - Inadequate discussion of theoretical or conceptual underpinning - Inadequate description of research setting and target population - Inadequate sampling - No evidence that research stakeholders were considered in the research design |
| Schultz (2021) | Australia | Mixed-method observational study | Survey | Virtual ward established during  the COVID-19 pandemic in a large metropolitan health service  in Australia | 1)140 patients and one carer. Respondents had a broad age range (18–84 years) with an equal distribution of male and female  2)17 virtual ward staff employes (10 nurses, one Allied Health staff member and six Administration Officers) | 158 participants (140 patients and 17 healthcare providers) | Covid-19 | The virtual ward staffing profile varied by demand and included Administrative Officers, Allied Health staff (Pharmacy and Social Work), Nursing staff and Medical Officers. At initial clinical assessment, the interval for telephone monitoring was established through a risk assessment checklist with individual’s receiving daily or twice daily calls. This could be modified based on subsequent consultations and risk reassessment Patients with scores of > 12 were referred to a  Medical Officer for review, as per management of the deteriorating patient pathway | 18 points   - Inadequate sampling - No rational for choice of data collection tool - No recruitment data provided - No justification of analytic method selected - No evidence that research stakeholders were considered in the research design - No discussion of strengths and limitations |
| Vindrola-Padros(2021) | England | Mixed-methods study | Interviews | 8 different sites: 2 Secondary care (ED) (prehospital), 2 Primary care (pre-hospital), 2 Secondary care (pre-hospital and  early discharge from the  hospital), 1 Secondary care (early discharge  from the hospital model), 1 Secondary care (early discharge  from the hospital model),  planning pre-hospital model | 1) 8 pilot site leads  2) 7 staff members in charge of monitoring  3) 7 staff members with knowledge of data collection/use | 22 participants (8 administrative staff members and 14 healthcare providers) | Covid-19 | The patient was provided with a pulse oximeter, patient information (including escalation warning signs and what to do) and a mechanism for recording observations regularly (app or paper diary) such as changes in symptoms, pulse, heart rate, temperature and blood oxygen levels. The patient received regular monitoring calls from staff (either primary or secondary care depending on the site) capturing changes in symptoms and trends in oxygen saturation. The apps had an additional safety mechanism, where these alerted the patient when recorded data indicated potential deterioration and the patient was instructed about next steps (emergency telephone numbers, GP call, etc.). Alerts could also trigger action by the clinical team monitoring patient information on the electronic dashboards, mainly through a phone call to the patient to indicate that they would need to visit a local ED or dial the emergency phone line. Patients were expected to be discharged from remote home monitoring around 14 days. | 21 points   - Inadequate theoretical underpinning - No rationale for data collection choice - Inadequate recruitment data - Inadequate justification of selected analytical method - No evidence that research stakeholders were considered in the research design - No discussion of strengths and limitations |
| Walton (2022) | UK | Mixed- methods study | Interviews | 17 different NHS trusts or primary care practices/Commissioning Groups (CCGs) that implemented COVID‐19 remote home monitoring services. | Patients who had received COVID‐19 remote home monitoring services were recruited into the survey. If patients were unable to take part but wanted to participate, we invited their carer to complete the survey/interview on their behalf. | 62 participants (59 patients and 3 family members) | Covid-19 | Patients are given a pulse oximeter, together with information and resources outlining how to use the equipment, escalation warning signs and what to do if these warning signs appear. Patients measure their oxygen saturation levels using the oximeter and other readings (pulse/heart rate/ temperature) regularly and record and submit these readings. Readings are shared by telephone or using a tech‐enabled method (e.g., an app on the patient's phone or computer). Patients are then escalated for further care if necessary. Discharge from the service is typically around 14 days. | 32 points   - Inadequate theoretical underpinning |
