# Supplementary material for: Barriers and facilitators to the use of virtual wards: a systematic review of the qualitative evidence
Source: Int J Qual Health Care. 2025 Jul 18;37(3):mzaf065. doi: 10.1093/intqhc/mzaf065 (PMC12342918; doi:10.1093/intqhc/mzaf065)
Supplement: mzaf065_Supplementary_Data [file mzaf065_supplementary_data.zip › INTQHC-2025-02-0056.R2_FINAL THEME MAPPING (Supplementary material 4a).docx]

**Supplementary Material 4a: Descriptive theme mapping to TDF Model**

| **COM-B domain** | **TDF domain** | **Descriptive theme** | **Author/s** | **Barriers** | **Author/s** | **Facilitators** | **Tot** |
| --- | --- | --- | --- | --- | --- | --- | --- |
| **CAPABILITY** | **Knowledge** | **Knowledge Gathering** | (Vindrola-Padros, 2021) (Schultz, 2021) | **2** | (Gagnon, 2020) (Harel, 2024) (Herlitz, 2023) | **3** | **5** |
|  |  | **Patient and Family Knowledge** | (Cerdan de Las Heras, 2023) (Ko a, 2023) (Vindrola-Padros, 2021) (Jessup, 2022) (Ko ,2023 b) (Ravi,2024) (Rodgers, 2012) (Walton, 2022) | **8** | (Asabo, 2024) (Kirckaldy, 2018) (Cerdan de Las Heras, 2023) (Walton, 2022) (Herltz, 2023) (Kirckaldy, 2018) (Rodgers, 2012) | **7** | **11** |
|  |  | **Knowledgeable Staff** | (Ravi, 2024) (Cerdan de Las Heras,2023) (Walton, 2022) (Ko, 2023 b) | **4** | (Rodgers, 2012) (Eines, 2023) (Jessup, 2022) (Gagnon, 2020) (Cerdan de Las Heras, 2023) (Harel, 2024) (Lee,2022) (Kirckaldy, 2018) (Ko, 2023 b) (Ravi, 2024) (Walton, 2022) | **11** | **11** |
|  |  | **Eligibility Criteria/Procedural Guidelines** | (Harel, 2024) (Vindrola-Padros, 2021) (Asabo, 2024) (Cerdan de Las Heras, 2023) (Kirckaldy, 2018) (Ko, 2023 a) (Ravi, 2024) | **7** | (Lee, 2022) (Asabo, 2024) (Cerdan de Las Heras, 2023) (Herlitz, 2023) (Jessup, 2022) | **5** | **10** |
|  |  | **Educational Resources** | (Ravi, 2024) | **1** | (Ravi, 2024) (Rodgers, 2012) (Vindrola-Padros, 2021) | **3** | **3** |
|  | **Skills** | **Competence/Experience** | (Rodgers, 2012) (Walton, 2022) | **2** | (Gagnon, 2020) (Asabo, 2024) (Kirckaldy, 2018) (Schultz, 2021) (Eines, 2023) | **5** | **7** |
|  |  | **Communication** | (Walton, 2022) (Asabo, 2024) (Ravi, 2024) | **3** | (Ko b, 2023) (Vindrola-Padros, 2021) (Cerdan de Las Heras, 2023) (Jessup, 2022) | **4** | **6** |
|  |  | **Training/Learning** |  | **0** | (Lee, 2022) (Asabo, 2024) (Cerdan de Las Heras, 2023) (Herlitz, 2023) (Vindrola-Padros, 2021) (Kirckaldy, 2018) | **6** | **6** |
|  |  | **Assessment accuracy** | (Lee, 2022) (Asabo, 2024) (Cerdan de Las Heras, 2023) (Harel, 2024) | **4** |  | **0** | **4** |
|  | **Behavioural regulation** | **Self-monitoring** | (Cerdan de Las Heras, 2023) | **1** | (Cerdan de Las Heras, 2023) | **1** | **1** |
|  |  | **Action planning** |  | **0** | (Walton, 2022) | **1** | **1** |
|  | **Memory, attention and decision process** | **Poor health/Tiredness** | (Walton, 2022) (Herlitz, 2023) (Vindrola-Padros, 2021) (Cerdan de Las Heras, 2023) (Kirckaldy, 2018) | **5** |  | **0** | **5** |
|  |  | **Cognitive capacities** | (Walton, 2022) (Cerdan de Las Heras, 2023) (Herlitz, 2023) (Jessup, 2022) (Kirckaldy, 2018) (Lee, 2022) (Ko, 2023 a) (Vindrola-Padros, 2021) | **8** | (Cerdan de Las Heras, 2023) (Walton, 2022) | **2** | **8** |
| **OPPORTUNITY** | **Environmental context and resources** | **Mixed models with flexible components** |  | **0** | (Gagnon, 2020) (Jessup, 2022) (Herlitz, 2023) (Schultz, 2021) | **4** | **4** |
|  |  | **Resources** | (Ravi, 2024) (Walton, 2022) (Lee, 2022) (Asabo, 2024) (Cerdan de Las Heras, 2023) (Harel, 2024) (Vindrola-Padros, 2021) (Jessup, 2022) (Ko, 2023 b) (Rodgers, 2012) (Schultz, 2021) | **11** | (Gagnon, 2020) (Ravi, 2024) (Walton, 2022) (Cerdan de Las Heras, 2023) (Jessup, 2022) (Ko, 2023 b) (Vindrola-Padros, 2021) (Asabo, 2024) (Eines, 2024) (Harel, 2024) (Lee, 2022) (Ko, 2023 a) (Schultz, 2021) | **13** | **14** |
|  |  | **Standardized protocols** | (Walton, 2022) (Harel, 2024) (Vindrola-Padros, 2021) | **3** | (Gagnon, 2020) (Lee, 2022) (Harel, 2024) (Jessup, 2022) (Ko, 2023 b) (Schultz, 2021) | **6** | **9** |
|  |  | **Technical/technology use** | (Walton, 2022) (Lee, 2022) (Ravi, 2024) (Cerdan de Las Heras, 2023) (Herlitz, 2023) (Ko, 2023 b) (Vindrola-Padros, 2021) (Schultz, 2021) (Kirkcaldy, 2018) | **9** | (Walton, 2022) (Cerdan de Las Heras, 2023) (Herlitz, 2023) (Schultz, 2021) (Eines, 2024) (Ko, 2023 b) (Ravi, 2024) | **7** | **10** |
|  | **Social influences** | **Positive relationship** |  | **0** | (Gagnon, 2020) (Lee, 2022) (Ravi, 2024) (Eines, 2023) (Herlitz, 2023) (Jessup, 2022) (Ko, 2023 a) (Rodgers, 2012) (Schultz, 2021) (Walton, 2022) | **10** | **10** |
|  |  | **Communication channels** | (Walton, 2022) (Asabo, 2024) (Cerdan de Las Heras, 2023) | **3** | (Gagnon, 2020) (Asabo, 2024) (Cerdan de Las Heras, 2023) (Jessup, 2022) | **4** | **5** |
|  |  | **Interprofessional work** |  | **0** | (Eines, 2023) (Kirkcaldy, 2018) (Lee, 2022) (Ravi, 2024) | **4** | **4** |
|  |  | **Social support** | (Lee, 2022) (Walton, 2022) (Ko, 2023 a) (Asabo, 2024) (Ko, 2023 b) | **5** | (Lee, 2022) (Walton, 2022) (Cerdan de Las Heras, 2023) (Eines, 2023) (Herlitz, 2023) (Ko, 2023 b) (Asabo, 2024) (Jessup, 2022) (Kirkcaldy, 2018) (Ko, 2023 a) (Ravi, 2024) | **11** | **11** |
| **MOTIVATION** | **Social/Professional Role & Identity** | **Stakeholders support** | (Ravi, 2024) (Vindrola-Padros, 2021) | **2** | (Eines, 2023) (Jessup, 2022) (Schultz, 2021) (Vindrola-Padros, 2021) (Gagnon, 2020) (Harel, 2024) | **6** | **7** |
|  |  | **Established healthcare professionals** |  | **0** | (Rodgers, 2012) (Ravi, 2024) (Kirckaldy, 2018) (Gagnon, 2020) (Cerdan de Las Heras, 2023) | **5** | **5** |
|  |  | **Adapting to new roles** | (Lee, 2022) (Cerdan de Las Heras, 2023) (Gagnon, 2020) (Harel, 2024) (Ko, 2023 b) (Ko, 2023 a) | **6** | (Cerdan de Las Heras, 2023) (Lee, 2022) | **2** | **6** |
|  |  | **Societal pressure** |  | **0** | (Ko, 2023 b) | **1** | **1** |
|  | **Emotions** | **Dis/Comfort** | (Walton, 2022) (Ko, 2023 b) | **2** | (Rodgers, 2012) (Asabo, 2024) (Kirckaldy, 2018) (Ko, 2023 a) (Cerdan de Las Heras, 2023) (Harel, 2024) (Ko, 2023 b) (Vindrola-Padros, 2021) | **8** | **9** |
|  |  | **Feeling important** |  | **0** | (Rodgers, 2012) (Jessup, 2022) | **2** | **2** |
|  |  | **Caregivers Burden/Burn-out** | (Lee, 2022) (Cerdan de Las Heras, 2023) (Harel, 2024) (Ko, 2023 b) | **4** |  | **0** | 4 |
|  |  | **Anxiety/stress** | (Walton, 2022) (Asabo, 2024) (Cerdan de Las Heras, 2023) (Jessup, 2022) (Lee, 2022) (Vindrola-Padros, 2021) | **6** |  | **0** | **6** |
|  | **Beliefs about consequences** | **Convenient and reliable care expectations** |  | **0** | (Lee, 2022) (Ko, 2023 a) (Walton, 2022) (Rodgers, 2012) (Cerdan de las Heras, 2023) (Kirckaldy, 2018) | **6** | **6** |
|  |  | **Avoiding negative consequences** | (Asabo, 2024) (Cerdan de Las Heras, 2023) (Lee, 2022) (Walton, 2022) | **4** | (Lee, 2022) (Walton, 2022) (Jessup, 2022) (Ko, 2023 a) | **4** | **6** |
|  |  | **Expected loss of control** | (Asabo, 2024) (Cerdan de Las Heras, 2023) (Harel, 2024)  (Ravi, 2024) (Walton, 2022) | **5** |  | **0** | **6** |
|  | **Reinforcement** | **Incentives** |  | **0** | (Walton, 2022) (Cerdan de Las Heras, 2023) (Harel, 2024) (Ko, 2023 a) (Jessup, 2022) (Lee, 2022) (Rodgers, 2012) | **7** | **7** |
|  |  | **Prompts** | (Walton, 2022) | **1** | (Walton, 2022) (Cerdan de Las Heras, 2023) (Ko, 2023 a) (Ko, 2023 b) | **4** | **4** |
|  |  | **Feedback** |  | **0** | (Walton, 2022) (Asabo, 2024) (Cerdan de Las Heras) (Herlitz, 2023) (Ko, 2023 b) (Rodgers, 2012) | **6** | **6** |
|  | **Beliefs about capabilities** | **Empowerment** | (Ravi, 2024) (Asabo, 2024) | **2** | (Rodgers, 2012) (Ravi, 2024) (Cerdan de Las Heras, 2023) (Eines, 2023) (Jessup, 2022) | **5** | **6** |
|  |  | **Perceived vulnerability** | (Rodgers, 2012) (Ravi, 2024) | **2** |  | **0** | **2** |
|  |  | **Perceived behavioural control** | (Cerdan de Las Heras, 2023) (Herlitz, 2023) (Jessup, 2022) (Ko, 2023 b) (Asabo, 2024) | **5** | (Jessup, 2022) (Asabo, 2024) (Cerdan de Las Heras, 2023) | **3** | **5** |
|  | **Goals** | **Evaluation research** |  | **0** | (Gagnon, 2020) | **1** | **1** |
|  |  | **Health seeking behaviour** |  | **0** | (Asabo, 2024) (Eines, 2023) (Cerdan de Las Heras, 2023) | **3** | **3** |
|  |  | **Meeting patients’ needs** |  | **0** | (Eines, 2023) (Cerdan de Las Heras, 2023) | **2** | **2** |
|  |  | **Agile Implementation** |  | **0** | (Schultz, 2021) | **1** | **1** |
|  | **Optimism** | **High Expectations** | (Kirckaldy, 2018) (Cerdan de Las Heras, 2023) | **2** |  | **0** | **1** |
|  |  | **Faith** |  | **0** | (Rodgers, 2012) | **1** | **1** |
|  | **Intentions** | **Building relationships** |  | **0** | (Rodgers, 2012) (Eines, 2023) (Cerdan de Las Heras, 2023) | **3** | **3** |
|  |  | **Discipline** | (Asabo, 2024) (Herlitz, 2023) (Lee, 2022) (Walton, 2022) (Ravi, 2024) (Ko, 2023 b) (Ko, 2023 a) (Eines, 2023) | **8** | (Lee, 2022) (Asabo, 2024) (Jessup, 2022) (Schultz, 2021) | **4** | **10** |
|  |  | **Clashing with everyday life** | (Cerdan de Las Heras, 2023) | **1** |  | **0** | **1** |
